# Incorporating *Euglena gracilis* into the habitual diet modulates immune function and reduces common cold-like symptoms: a randomized controlled trial

**DOI:** 10.1186/s12937-026-01350-6

**Published:** 2026-06-09

**Authors:** Ayaka Nakashima, Wataru Okamoto, Yuka Marukawa-Hashimoto, Tsuyoshi Takara

**Affiliations:** 1 Euglena Co., Ltd., G-BASE Tamachi 2F, 5-29-11, Shiba, Minato-ku, Tokyo, 108-0014 Japan; 2 Medical Corporation Seishinkai, Takara Clinic., 9F Taisei Building, 2-3-2 Higashi-gotanda, Shinagawa-ku, Tokyo, 141-0022 Japan

**Keywords:** *Euglena gracilis*, Paramylon, Plasmacytoid dendritic cells, Cold symptoms, Immune markers

## Abstract

**Background:**

Dietary approaches to support immune function have emphasized the importance of dietary fiber, including non-digestible polysaccharides, although intake remains below recommended levels in many populations. In this context, microalgae have attracted attention as sustainable sources of these components. *Euglena gracilis*, a microalga used in foods, contains paramylon, a β-1,3-glucan with reported immunomodulatory properties; however, its effects on plasmacytoid dendritic cell (pDC) activation in humans within a dietary context remain incompletely understood.

**Methods:**

This randomized, double-blind, placebo-controlled trial investigated the effects of 8-week *E. gracilis* intake (1,000 mg/day) on immune function and common cold symptoms in healthy Japanese adults. A total of 200 participants were randomized, of whom 181 (91 in the *Euglena* group and 90 in the placebo group) were included in the per-protocol analysis. The primary endpoint was the change in the log-transformed median fluorescence intensity (MFI) of CD86 on pDCs at Week 8.

**Results:**

Analysis using a linear mixed model revealed that the mean ± standard deviation of the log-transformed CD86 MFI at Week 8 was higher in the *Euglena* group than in the placebo group (placebo: 7.0 ± 2.1; *Euglena*: 7.7 ± 0.4; group difference 0.7; 95% confidence interval 0.2–1.1; *P* = 0.004). As exploratory secondary outcomes, the circulating monocyte percentage was higher and the incidence of common cold symptoms—including chills, fatigue, sneezing, rhinorrhea, nasal congestion, sore throat, cough, and headache—was lower in the *Euglena* group than in the placebo group.

**Conclusions:**

These findings suggest that daily consumption of *E. gracilis* may support immune function and be associated with a reduced incidence of common cold-like symptoms. The intervention was well tolerated, with no adverse events reported and no clinically meaningful changes in hematological or urinalysis parameters. These findings support the potential of *E. gracilis* consumption as a viable dietary strategy.

**Trial registration:**

UMIN Clinical Trials Registry, UMIN000056125.

**Supplementary Information:**

The online version contains supplementary material available at 10.1186/s12937-026-01350-6.

## Introduction

Dietary patterns and specific food components are recognized as key modulators of immune function and homeostasis [1]. Dietary fibers, including non-digestible polysaccharides, regulate host defense mechanisms [2, 3]. However, dietary fiber intake remains below the recommended levels in many populations, highlighting the need to expand available dietary sources. In this context, microalgae have attracted interest as sustainable sources of functional dietary components. The microalga *Euglena gracilis* (*E. gracilis*), incorporated into foods and dietary practices [4], is a natural source of β-glucan because it contains paramylon—a linear β-1,3-glucan—as its principal component, along with nutrients such as vitamins, minerals, amino acids, and lipids.

Paramylon and paramylon-rich *E. gracilis* preparations have been reported to influence immune-related outcomes, including enhanced host defense against viral infection in mice, immune cell activation in humans, and modulation of immune responses in several animal models [5–10]. Clinical trials have shown that *E. gracilis* intake reduces common cold symptoms [11–13]. In addition, biomarker-focused clinical studies have reported increases in natural killer cell activity and salivary immunoglobulin A secretion after *E. gracilis* intake [14, 15], as well as maintenance of immune activation markers, such as CD80 and CD28, on monocytes and T cells [12]. These findings, observed across generally healthy adult populations, suggest that *E. gracilis* may support immune function and resilience within a dietary context.

Dendritic cells (DCs) play a pivotal role in coordinating innate and adaptive immunity [16–18]. Among DC subsets, plasmacytoid dendritic cells (pDCs) are specialized for antiviral defense and are considered indicators of immune readiness in humans [19]. β-glucans such as paramylon interact with Dectin-1, a pattern recognition receptor expressed on antigen-presenting cells including DCs, and modulate immune responses in experimental models [20–26]. However, previous studies have focused on macrophages [27], monocytes [12, 28], and neutrophils [28], and the effects on pDCs remain poorly understood. To our knowledge, no clinical study has evaluated the effects of *E. gracilis* on pDC activation in humans.

The present study was designed to evaluate the effects of *E. gracilis* intake on pDC activation in healthy Japanese adults within a dietary intervention framework. The primary endpoint was the median fluorescence intensity (MFI) of CD86 on pDCs at Week 8, indicating activation status. Secondary endpoints included CD86 MFI at Week 4, postintervention HLA-DR MFI on pDCs, circulating immune cell subset proportions, allergen-specific IgE levels, bowel movement parameters, safety outcomes, and the incidence of common cold symptoms and individual symptoms. Common cold symptoms were assessed as a supportive clinical measure to determine whether changes in pDC activation were accompanied by clinical outcomes and to evaluate the reproducibility of prior clinical findings [11–13].

## Materials and methods

### Food materials

*E. gracilis* powder, derived from the *E. gracilis* Z strain, was obtained from Euglena Co., Ltd. (Tokyo, Japan) and encapsulated as the active test food. The powder consisted of dried *E. gracilis* cells that were nonviable due to the drying process. Meanwhile, the placebo capsules contained identical excipients, substituting partially pregelatinized starch for *E. gracilis* powder.

Both foods were provided as indistinguishable hard capsules in terms of appearance, taste, and odor. Each active capsule contained 250 mg of *E. gracilis* powder along with excipients, whereas the placebo capsules contained identical excipients without *E. gracilis* powder, ensuring comparable composition apart from the active ingredient.

Each test food was assigned an identification code, and the allocation list was securely stored and concealed from all study personnel until study completion, ensuring blinding throughout the study.

### Study design and participants

#### Target demographics

Eligible participants were healthy Japanese men and women aged ≥ 20 years who had experienced at least one episode of common cold symptoms during the same season as the intervention period (February–April) within the past 2 years. Detailed exclusion criteria are provided in the Supplementary Methods.

Participants were recruited via Go106 (https://www.go106.jp/), an online platform, with additional announcements on affiliated social networking platforms. Eligible individuals were required to attend all scheduled visits at the designated clinic in Tokyo and provide informed consent electronically. No participants affiliated with the study sponsor or funding organization were included. Participants received compensation in accordance with their compliance with the study protocol. Participants were not involved in the design, conduct, reporting, or dissemination plans of this study.

The sample size was determined based on a two-sided significance level of 5%, a power of 90%, equal allocation between the groups, and a two-group comparison, with the primary outcome set to detect a standardized effect size of 0.5 [29]. Under these conditions, at least 86 participants per group (172 in total) were required. To further ensure the robustness of the results, the target sample size was increased to 90 participants per group (180 in total). Furthermore, to account for potential dropouts during the trial period, an additional 10 participants per group were enrolled, yielding a total of 200 participants.

Participant recruitment was conducted from November 6, 2024, to December 22, 2024, with the first participant enrolled on November 13, 2024. The overall trial period extended from November 13, 2024, to April 27, 2025.

#### Trial methods

This trial was a single-center, randomized, double-blind, placebo-controlled, parallel-group, trial. The protocol was approved by the Ethics Committee of the Takara Clinic, Medical Corporation Seishinkai (Tokyo, Japan), on October 30, 2024 (approval no. 2410-00395-0045–1 C-TC) and was prospectively registered in the UMIN Clinical Trials Registry on November 12, 2024 (UMIN000056125; https://center6.umin.ac.jp/cgi-open-bin/ctr_e/ctr_view.cgi?recptno=R000064130). The trial conformed to the principles of the Declaration of Helsinki and the Ethical Guidelines for Medical and Biological Research Involving Human Subjects in Japan. No protocol modifications were made following registration.

### Outcome measures

#### Primary outcome

DC subsets were identified using the lineage depletion strategy described by Olweus et al. [30]. pDCs were defined as Lineage^−^ HLA-DR^+^ CD11c^−^ CD123^+^ cells [31]. CD86 expression was analyzed by flow cytometry (Navios EX, Beckman Coulter), and the primary endpoint was predefined as the log-transformed CD86 MFI within the gated pDC population at Week 8. Detailed blood processing, staining procedures, gating strategy, and instrument settings are described in the Supplementary Methods.

#### Secondary outcomes

##### Immune-related indices

Prespecified secondary immune-related endpoints included the log-transformed MFI of CD86 on pDCs at Week 4, the log-transformed MFI of HLA-DR on pDCs at Week 4 and Week 8, quantified within the same gated pDC population. Additional endpoints included peripheral leukocyte differential percentages (neutrophils, lymphocytes, monocytes, eosinophils, and basophils), and allergen-specific immunoglobulin E (IgE; cedar pollen and cypress pollen) levels measured at Week 4 and Week 8.

##### Common cold symptom survey

Common cold symptoms were self-reported by participants through a daily electronic diary and were not confirmed by a physician diagnosis. Throughout the intervention period, a prespecified common cold symptom survey was conducted. Outcomes were summarized for the overall period, Weeks 1–4 (first half) and Weeks 5–8 (second half), to evaluate clinical relevance, the reproducibility of previous findings, and symptom dynamics within the same population in which immune indices were evaluated. A total of 12 symptoms were assessed: (1) general malaise, (2) chills, (3) feverishness, (4) fatigue, (5) sneezing, (6) runny nose, (7) nasal congestion, (8) sore throat, (9) coughing, (10) arthralgia, (11) myalgia, and (12) headache. Each symptom was rated on a 5-point scale (1 = none, 2 = almost none, 3 = mild, 4 = moderate, 5 = severe). A response of 3–5 for a given symptom was classified as “symptom present,” and the number of participants reporting each symptom was documented. A participant was considered to have developed “common cold symptoms” if at least 1 of the 12 symptoms was rated 3–5 at any time during the evaluation period. The number of such participants during the intervention period was used for the analysis.

##### Bowel movement survey

Bowel movement outcomes were evaluated as exploratory gastrointestinal outcomes as *E. gracilis* contains non-digestible components, including paramylon, and previous studies have demonstrated that *E. gracilis* may influence bowel habits [32]. These outcomes were not intended to directly test the primary immune-related hypothesis. Bowel movement outcomes included weekly bowel movement frequency, number of bowel movement days, and stool volume (weekly sum), assessed from the week preceding the intervention initiation to Week 8. Frequency and number of days were derived from daily diaries. Stool volume was evaluated using a 20-mL film canister (diameter: 26 mm; height: 54.5 mm; total height: 57.0 mm) as a reference. The film canister was used as a simple visual reference to help participants consistently estimate stool volume in a home-based diary setting. Stool form was classified into eight predefined types (1 = watery diarrhea to 8 = pellet-like), adapted from the stool form categories used in the bowel function assessment described by Davies et al. [33]. For each participant and evaluation period, the median daily stool form score over the week was calculated and rounded to the nearest integer to define the representative stool form classification. Subsequently, the number of participants in each classification was tabulated.

##### Safety evaluation

Safety evaluations included physical measurements, urinalysis, and peripheral blood tests. Body weight, body mass index (BMI), and systolic/diastolic blood pressure were measured at screening, Week 4, and Week 8. Height was determined at screening and used for BMI calculation. Urinalysis and peripheral blood laboratory parameters were evaluated by LSI Medience Corporation (Tokyo, Japan) using standardized clinical laboratory methods. Adverse events (AEs) were defined as any new clinically meaningful abnormality or worsening of existing signs/symptoms during the trial. Minor gastrointestinal and transient symptoms were also recorded as AEs when they represented new clinically meaningful abnormalities or worsening of existing symptoms, in accordance with the predefined AE criteria. Upon AE onset, the principal investigator promptly provided appropriate care, determined whether the trial should continue and unblinding was necessary, and assessed the AE’s causality with the test food. The participants’ health status, compliance with test food intake, medication use, and lifestyle factors were recorded daily in a study diary and reviewed at each visit.

### Randomization

During screening, eligible candidates were randomized in a 1:1 ratio to the *Euglena* or placebo group by an allocation manager. The assignment list was generated with R (version 4.4.2) using the blockrand package (version 1.5), employing stratified block randomization with random block sizes. Stratification factors included sex, age, and CD86 MFI.

### Allocation and blinding

The allocation manager prepared the assignment order, assignment list, and emergency keys based on food identification numbers. Each emergency key was sealed in an envelope per participant, with the sealing date recorded and a security seal affixed. The assignment list and emergency keys were stored under strict control, and the former was not provided to the sponsor until the trial conclusion. After the trial was completed and the database was locked, the allocation manager confirmed that neither the assignment list nor the emergency keys had been accessed. The opening date and signature were recorded on the assignment list. Subsequently, the food identification codes were unblinded. Throughout the trial, blinding was maintained for all parties other than the allocation manager.

### Participant procedures

The trial schedule is presented in Supplementary Table 1. Baseline assessments were conducted at screening prior to the intervention initiation. Physical measurements, urinalysis, and peripheral blood tests were conducted during screening. Venous blood samples were collected in a seated position. The participants were instructed to fast for at least 6 h before blood collection. Water intake was allowed during the fasting period. The time of blood sampling was not fixed and was scheduled based on participant availability. No adjustment was made for the menstrual cycle phase. Samples were immediately processed, and aliquots were stored at appropriate temperatures until analysis. Blood collection was performed to assess immunological markers and clinical safety parameters.

After the baseline assessments, the participants began consuming the assigned test food and recording daily symptoms and bowel movements throughout the intervention period. They consumed four capsules per day of either *Euglena*-containing food or placebo for 8 weeks. The intervention period (February–April) was set to match that of the previous study [13]. The participants were instructed to take the capsules in the morning. If a dose was missed, they were allowed to take it later the same day. Furthermore, they were required to report their daily intake status to study staff via the electronic diary.

Surveys regarding common cold symptoms and bowel movements were conducted throughout the intervention period. The participants recorded daily the presence or absence of common cold symptoms. For bowel movements, they made entries at each event. They returned to the clinic at Week 4 and Week 8 for follow-up visits, during which the same assessments conducted at baseline were repeated.

AEs were monitored throughout the intervention. Specifically, AEs were identified based on participant self-reports. The participants were instructed to immediately contact the study staff if they experienced any changes in their physical condition. Upon receiving such reports, the study staff promptly notified the principal investigator, and appropriate follow-up measures were undertaken according to the predefined safety management procedures.

### Participant compliance

The participants were instructed to strictly adhere to the following compliance requirements throughout the trial period:

- (1) Take the assigned test food according to the prescribed dosage and schedule. (2) Maintain an intake rate of ≥ 80% throughout the trial period. (3) Ensure an intake rate of 100% in the week preceding the Week 8 examination. (4) Avoid overeating or overdrinking and refrain from changing usual lifestyle habits from consent to final examination. (5) Abstain from alcohol consumption and excessive physical activity from the day before each examination until its completion. (6) Immediately report any health changes during the study period to the study staff and follow their instructions. (7) As much as possible, avoid immune-modulating supplements, foods or beverages labeled as FOSHU or FFC, as well as products containing ingredients with potential functional or physiological effects. (8) If medications potentially affecting immune function (e.g., cold remedies, analgesics) were required within 3 days prior to the examination, reschedule the examination. (9) Refrain from receiving vaccinations for infectious disease prevention during the study period, unless unavoidable due to public health circumstances.

Participants were also instructed to maintain their regular dietary patterns throughout the trial period. Dietary intake, including dietary fiber intake, was monitored using dietary records [34] for the 3 days preceding each examination to determine whether substantial changes in dietary habits occurred during the intervention.

Compliance was monitored using a daily electronic diary, through which participants reported their intake of the assigned intervention food to the study staff. Intake compliance was determined by the proportion of capsules consumed relative to the prescribed dose.

### Institutions

The implementing institution, Medical Corporation Seishinkai, Takara Clinic (Tokyo, Japan), oversaw data evaluation and participant health management. Clinical testing procedures were performed in collaboration with the affiliated medical institution, Nerima Medical Association, Minami-machi Clinic (Tokyo, Japan).

### Statistical analysis

After trial completion, the principal investigator held a case review meeting to evaluate protocol adherence, eligibility compliance, consent irregularities, protocol deviations, and withdrawals. Data acceptance and the statistical analysis plan were finalized under blinded conditions before the database was locked.

The primary endpoint was prespecified as the log-transformed CD86 MFI on pDCs at Week 8. Log transformation was defined a priori in the statistical analysis plan, as fluorescence intensity measurements are frequently analyzed on the logarithmic scale.

The secondary endpoints included the log-transformed MFI of CD86 on pDCs at Week 4, the log-transformed MFI of HLA-DR on pDCs at Week 4 and Week 8, peripheral leukocyte differential percentages, allergen-specific IgE levels, incidence of common cold symptoms and individual symptoms, bowel movement parameters, and safety outcomes.

In this study, a repeated-measures design was implemented. Efficacy assessments (except for the common cold symptom and bowel movement surveys) were conducted at screening (baseline), Week 4, and Week 8. For the common cold symptom survey, evaluation periods were prespecified as the overall intervention period, the first half (Weeks 1–4), and the second half (Weeks 5–8). For the bowel movement survey, evaluation periods were defined as the week before screening (during the 7 day preceding the screening visit; period 0) and the subsequent weeks during the intervention (periods 1–8).

The intention-to-treat (ITT) population consisted of all randomized participants. The full analysis set (FAS) was defined as the ITT population excluding prespecified cases: those who did not receive the allocated intervention, those who did not meet the target population criteria, those who never received the intervention following allocation, and those who had no post-randomization data. The per-protocol set (PPS) was defined as the FAS excluding participants with major protocol deviations, such as < 80% ingestion, substantial compliance violations, intake of medications or foods plausibly influencing outcomes, notable lifestyle changes, or other predefined reasons compromising data reliability. According to the prespecified statistical analysis plan, the analysis populations (ITT, FAS, and PPS) were hierarchically defined, and predefined criteria were specified to determine the primary efficacy analysis population. The final analysis dataset was selected based on these criteria before the database was locked. In accordance with these prespecified rules, efficacy analyses were conducted using the PPS dataset. Safety outcomes were evaluated using the safety analysis set, defined as all randomized participants who received at least one dose of the randomized intervention.

Baseline characteristics were compared between the groups using Welch’s *t*-test for continuous variables. Baseline between-group differences for continuous variables were expressed as mean differences with corresponding 95% confidence intervals (CIs).

For continuous outcomes, immune-related endpoints were analyzed using linear mixed-effects models. The baseline value was included as a covariate. The fixed effects included the treatment group, visit (Week 4 and Week 8), treatment × visit interaction, and baseline × visit interaction. Each participant was included as a random intercept to account for within-subject correlation across repeated measurements. Data were expressed as mean ± standard deviation. Postintervention between-group differences were derived from the fitted model and were expressed as differences in estimated marginal means with corresponding 95% CIs. The prespecified primary comparison was the adjusted between-group difference at Week 8 derived from the model.

For categorical outcomes, including the incidence of common cold symptoms and categorical safety assessments, the number and proportion of applicable participants were reported. Between-group comparisons were performed using Pearson’s chi-squared test, and effect sizes were expressed as odds ratios with 95% CIs, alongside differences in proportions and their corresponding 95% CIs. For stool form classifications in the bowel movement survey, the number of participants corresponding to each stool form classification and the adjusted residuals were reported for each group, and between-group differences were evaluated using Pearson’s chi-squared test.

The primary endpoint was analyzed without multiplicity adjustment, as it represented the sole confirmatory hypothesis. Because the secondary endpoints were prespecified as exploratory and hypothesis-generating, no formal multiplicity adjustment was applied; therefore, findings from secondary outcomes should be interpreted cautiously.

All statistical tests were two-sided with a significance level of 5%. Continuous outcomes analyzed using linear mixed-effects models were estimated under a missing-at-random assumption inherent to the model. Missing data were not imputed. Owing to the low proportion of missing data and balanced discontinuations between groups, additional imputation-based sensitivity analyses were not conducted. For categorical outcomes and safety assessments, a complete case analysis was employed. Analyses were conducted using SPSS Statistics (version 23 or higher).

## Results

### Analysis population

Of the 264 individuals who provided informed consent, 200 met the eligibility criteria and were equally randomized to the *Euglena* (*n* = 100) and placebo (*n* = 100) groups; the remaining 64 were excluded for not meeting the eligibility criteria. All participants received the allocated intervention. However, one participant in the *Euglena* group failed to attend the Week 4 visit, seven (four in the *Euglena* group and three in the placebo group) failed to attend visits after week 4, and three participants (one in the *Euglena* group and two in the placebo group) failed to attend the week-8 visit. The seven participants who failed to return after Week 4 were confirmed not to have ingested the test food after allocation and were therefore excluded from the analyses.

**Fig. 1:**
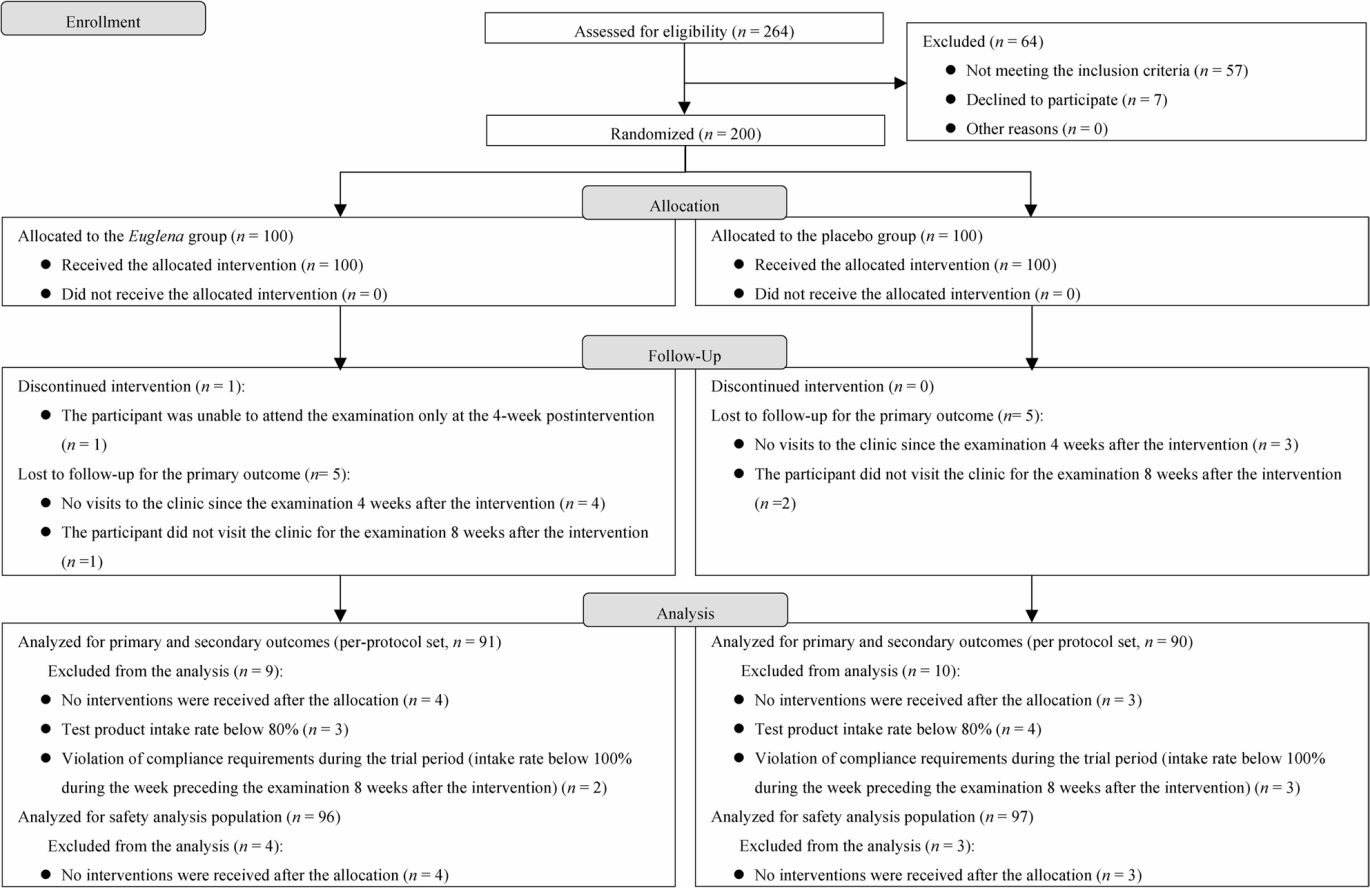
Participant flow

For efficacy analyses, in addition to the aforementioned exclusions, seven participants (three in the *Euglena* group and four in the placebo group) with test food intake rates < 80% and five participants (two in the *Euglena* group and three in the placebo group) who violated compliance requirements (intake rate < 100% during the week preceding the Week 8 assessment) were excluded. Consequently, the PPS comprised 181 participants (91 in the *Euglena* group and 90 in the placebo group, 20–82-year-old men and women). The SAF included 193 participants (96 in the *Euglena* group and 97 in the placebo group, 20–82-year-old men and women [Fig. 1]).

Participant demographics and baseline characteristics are presented in Supplementary Tables 2–4, and the presence of common cold symptoms during the past 2 years or within the week preceding the screening is summarized in Supplementary Table 5. No statistically significant differences were observed in baseline characteristics. The mean (minimum–maximum) test food intake compliance during the intervention period was 99.7% (94.6–100.0%) in both groups. During the intervention period, dietary records were reviewed, and no apparent between-group imbalances were identified in the intake of energy or nutrients, including dietary fiber.

### Immune indicators

The MFI of CD86 and HLA-DR on pDCs is presented in Table 1. The primary outcome—the log-transformed CD86 MFI on pDCs at Week 8—was 7.7 ± 0.4 (mean ± SD) in the *Euglena* group and 7.0 ± 2.1 in the placebo group. The estimated between-group difference was 0.7 (95% CI: 0.2–1.1; *P* = 0.004), with higher values observed in the *Euglena* group.

**Table 1:** Immunological indicators (PPS)

Item	Unit	Time point	*Euglena* group			Placebo group			Group difference			
			*n*	Mean	SD	*n*	Mean	SD	Δ	95% CIs−	95% CIs+	*P* value
pDC CD86 MFI	-	Screening	91	8.1	0.5	90	8.1	0.5	0.0	−0.1	0.1	0.988
		Week 4	90	7.7	1.0	90	7.3	1.8	0.4	−0.1	0.8	0.098
		Week 8	90	7.7	0.4	89	7.0	2.1	0.7	0.2	1.1	0.004**
pDC HLR-DR MFI	-	Screening	91	11.7	0.7	90	11.7	0.7	0.0	−0.2	0.2	0.729
		Week 4	90	11.8	0.5	90	11.7	0.5	0.0	−0.1	0.2	0.772
		Week 8	90	11.7	0.7	89	11.6	1.4	0.1	−0.2	0.5	0.475
Neutrophil percentage	%	Screening	91	59.8	8.5	90	58.2	7.5	1.6	−0.7	4.0	0.178
		Week 4	90	60.1	9.1	90	59.1	7.2	0.2	−1.9	2.3	0.827
		Week 8	90	59.6	8.8	89	58.3	8.3	0.3	−1.8	2.4	0.762
		Change (Week 4)	90	0.3	8.9	90	0.9	7.4	0.2	−1.9	2.3	0.827
		Change (Week 8)	90	−0.2	8.2	89	−0.1	7.2	0.3	−1.8	2.4	0.762
Lymphocyte percentage	%	Screening	91	31.1	8.2	90	32.5	7.3	−1.3	−3.6	0.9	0.249
		Week 4	90	30.2	8.3	90	31.5	6.6	−0.6	−2.5	1.2	0.506
		Week 8	90	31.2	8.5	89	32.5	7.8	−0.5	−2.4	1.4	0.610
		Change (Week 4)	90	−0.9	7.6	90	−0.9	6.8	−0.6	−2.5	1.2	0.506
		Change (Week 8)	90	0.0	7.3	89	0.2	6.5	−0.5	−2.4	1.4	0.610
Monocyte percentage	%	Screening	91	5.4	1.3	90	5.5	1.3	−0.1	−0.5	0.3	0.701
		Week 4	90	5.3	1.3	90	5.3	1.2	0.0	−0.3	0.3	0.821
		Week 8	90	5.5	1.4	89	5.3	1.3	0.3	0.0	0.6	0.033*
		Change (Week 4)	90	−0.1	1.2	90	−0.2	1.1	0.0	−0.3	0.3	0.821
		Change (Week 8)	90	0.1	1.1	89	−0.2	0.9	0.3	0.0	0.6	0.033*
Eosinophil percentage	%	Screening	91	2.8	2.3	90	2.9	2.4	−0.1	−0.8	0.6	0.764
		Week 4	90	3.5	2.9	90	3.1	2.3	0.4	−0.1	1.0	0.138
		Week 8	90	2.8	2.0	89	3.0	2.4	−0.1	−0.5	0.3	0.581
		Change (Week 4)	90	0.7	2.5	90	0.2	1.3	0.4	−0.1	1.0	0.138
		Change (Week 8)	90	0.0	1.6	89	0.2	1.5	−0.1	−0.5	0.3	0.581
Basophil percentage	%	Screening	91	0.8	0.4	90	0.9	0.4	−0.1	−0.2	0.0	0.084
		Week 4	90	0.9	0.4	90	0.9	0.5	0.0	−0.1	0.1	0.505
		Week 8	90	0.9	0.4	89	0.9	0.4	0.1	0.0	0.2	0.200
		Change (Week 4)	90	0.0	0.4	90	0.0	0.4	0.0	−0.1	0.1	0.505
		Change (Week 8)	90	0.0	0.4	89	−0.1	0.3	0.1	0.0	0.2	0.200

Data are summarized as the mean and standard deviation (SD). Between-group differences (Δ) at screening are expressed as differences in means. Post-intervention differences are derived from linear mixed models and are presented as differences in estimated marginal means with their corresponding 95% confidence intervals (CIs)

*pDC* plasmacytoid dendritic cell, *MFI* median fluorescence intensity, *HLA* human leukocyte antigen, *PPS* per protocol set, *Week 4* 4 weeks after intervention, *Week 8* 8 weeks after intervention, *Change* change from screening

*P < 0.05, **P < 0.01

The neutrophil, lymphocyte, monocyte, eosinophil, and basophil percentages are also shown in Table 1. At Week 8, the monocyte percentages were 5.5 ± 1.4% and 5.3 ± 1.3% (mean ± SD) in the *Euglena* and placebo groups, respectively, with corresponding changes from baseline of 0.1 ± 1.1% and − 0.2 ± 0.9% (mean ± SD). The estimated between-group difference was 0.3% (95% CI: 0.0–0.6; *P* = 0.033), with higher monocyte percentages observed in the *Euglena* group.

For the log-transformed MFI of CD86 on pDCs at Week 4, the MFI of HLA-DR on pDCs, and neutrophil, lymphocyte, eosinophil, and basophil percentages at all assessed time points, no significant differences were observed.

Similarly, no statistically significant between-group differences were observed in the proportion of participants showing an improvement of at least one grade in cedar- or cypress-specific IgE class at either Week 4 or Week 8 relative to baseline (Table 2).

**Table 2:** Participants who showed an improvement in the specific IgE class for cedar pollen or cypress pollen (PPS)

Item	Time point	*Euglena* group			Placebo group			Group difference						
		*n*	Number of applicable participants	Proportion (%)	*n*	Number of applicable participants	Proportion (%)	ORs	95% CIs−	95% CIs+	Δ (%)	95% CIs−	95% CIs+	*P* value
Participants whose cedar pollen–specific IgE (class) improved by at least one grade compared with the screening	Week 4	90	4	4.4	90	10	11.1	0.4	0.1	1.2	−6.7	−14.5	1.2	0.095
	Week 8	90	0	0.0	89	1	1.1	NA	NA	NA	−1.1	−3.3	1.1	0.313
Participants whose cypress pollen–specific IgE (class) improved by at least one grade compared with the screening	Week 4	90	5	5.6	90	10	11.1	0.5	0.2	1.4	−5.6	−13.6	2.5	0.178
	Week 8	90	2	2.2	89	2	2.2	1.0	0.1	7.2	0.0	−4.4	4.3	0.991

The number and proportion of applicable cases are reported, and between-group comparisons are presented as odds ratios (ORs) with 95% confidence intervals (CIs), along with differences in proportions (Δ) and their corresponding 95% CIs. Between-group comparisons were conducted using Pearson’s chi-square test

*PPS* per protocol set, *IgE* immunoglobulin E, *Week 4* 4 weeks after intervention, *Week 8* 8 weeks after intervention

### Common cold symptom survey

The number of participants who exhibited common cold symptoms is presented in Table 3.

**Table 3:** Participants who experienced each cold-like symptom during the study period (PPS)

Item	Period	*Euglena* group			Placebo group			Group difference						
		*n*	Number of applicable participants	Proportion (%)	*n*	Number of applicable participants	Proportion (%)	ORs	95% CIs−	95% CIs+	Δ (%)	95% CIs−	95% CIs+	*P* value
Number of participants with common cold symptoms	Overall	91	67	73.6	90	85	94.4	0.16	0.06	0.45	−20.8	−31.5	−10.1	< 0.001***
	1st half	91	63	69.2	90	82	91.1	0.22	0.09	0.51	−21.9	−33.5	−10.2	< 0.001***
	2st half	91	62	68.1	90	77	85.6	0.36	0.17	0.75	−17.4	−29.7	−5.1	0.005**
Number of participants with general malaise	Overall	91	45	49.5	90	54	60.0	0.65	0.36	1.18	−10.5	−25.1	4.0	0.154
	1st half	91	38	41.8	90	41	45.6	0.86	0.48	1.54	−3.8	−18.2	10.7	0.607
	2st half	91	36	39.6	90	40	44.4	0.82	0.45	1.48	−4.9	−19.3	9.5	0.506
Number of participants with chills	Overall	91	22	24.2	90	37	41.1	0.46	0.24	0.86	−16.9	−30.6	−3.3	0.015*
	1st half	91	17	18.7	90	32	35.6	0.42	0.21	0.82	−16.9	−29.8	−3.9	0.011*
	2st half	91	14	15.4	90	20	22.2	0.64	0.30	1.36	−6.8	−18.2	4.5	0.239
Number of participants with feverishness	Overall	91	15	16.5	90	25	27.8	0.51	0.25	1.05	−11.3	−23.4	0.8	0.067
	1st half	91	10	11.0	90	17	18.9	0.53	0.23	1.23	−7.9	−18.3	2.5	0.136
	2st half	91	12	13.2	90	14	15.6	0.82	0.36	1.90	−2.4	−12.6	7.9	0.650
Number of participants with fatigue	Overall	91	54	59.3	90	77	85.6	0.25	0.12	0.51	−26.2	−39.2	−13.2	< 0.001***
	1st half	91	49	53.8	90	64	71.1	0.47	0.26	0.88	−17.3	−31.4	−3.2	0.016*
	2st half	91	47	51.6	90	70	77.8	0.31	0.16	0.58	−26.1	−40.1	−12.2	< 0.001***
Number of participants with sneezing	Overall	91	46	50.5	90	56	62.2	0.62	0.34	1.12	−11.7	−26.1	2.8	0.113
	1st half	91	38	41.8	90	45	50.0	0.72	0.40	1.29	−8.2	−22.8	6.3	0.266
	2st half	91	32	35.2	90	45	50.0	0.54	0.30	0.98	−14.8	−29.2	−0.4	0.044*
Number of participants with a runny nose	Overall	91	48	52.7	90	64	71.1	0.45	0.25	0.84	−18.4	−32.5	−4.2	0.011*
	1st half	91	43	47.3	90	59	65.6	0.47	0.26	0.86	−18.3	−32.8	−3.9	0.013*
	2st half	91	35	38.5	90	43	47.8	0.68	0.38	1.23	−9.3	−23.7	5.1	0.206
Number of participants with nasal congestion	Overall	91	31	34.1	90	47	52.2	0.47	0.26	0.86	−18.2	−32.6	−3.7	0.014*
	1st half	91	23	25.3	90	40	44.4	0.42	0.23	0.79	−19.2	−33.0	−5.3	0.007**
	2st half	91	25	27.5	90	31	34.4	0.72	0.38	1.36	−7.0	−20.4	6.5	0.310
Number of participants with sore throat	Overall	91	30	33.0	90	45	50.0	0.49	0.27	0.90	−17.0	−31.4	−2.7	0.020*
	1st half	91	23	25.3	90	35	38.9	0.53	0.28	1.00	−13.6	−27.2	0.0	< 0.050*
	2st half	91	24	26.4	90	33	36.7	0.62	0.33	1.17	−10.3	−23.8	3.2	0.136
Number of participants with coughing	Overall	91	26	28.6	90	43	47.8	0.44	0.24	0.81	−19.2	−33.4	−5.1	0.008**
	1st half	91	21	23.1	90	35	38.9	0.47	0.25	0.90	−15.8	−29.3	−2.3	0.021*
	2st half	91	17	18.7	90	30	33.3	0.46	0.23	0.91	−14.7	−27.4	−1.9	0.025*
Number of participants with arthralgia	Overall	91	26	28.6	90	26	28.9	0.98	0.52	1.87	−0.3	−13.5	12.9	0.962
	1st half	91	19	20.9	90	22	24.4	0.82	0.41	1.64	−3.6	−15.8	8.6	0.567
	2st half	91	21	23.1	90	17	18.9	1.29	0.63	2.64	4.2	−7.7	16.1	0.489
Number of participants with myalgia	Overall	91	32	35.2	90	36	40.0	0.81	0.45	1.49	−4.8	−18.9	9.3	0.502
	1st half	91	23	25.3	90	28	31.1	0.75	0.39	1.43	−5.8	−18.9	7.3	0.383
	2st half	91	28	30.8	90	27	30.0	1.04	0.55	1.95	0.8	−12.6	14.2	0.910
Number of participants with headache	Overall	91	29	31.9	90	45	50.0	0.47	0.26	0.86	−18.1	−32.5	−3.8	0.013*
	1st half	91	22	24.2	90	35	38.9	0.50	0.26	0.95	−14.7	−28.2	−1.2	0.033*
	2st half	91	24	26.4	90	29	32.2	0.75	0.40	1.43	−5.8	−19.1	7.4	0.387

The number and proportion of applicable participants are reported, and between-group comparisons are presented as odds ratios (ORs) with 95% confidence intervals (CIs), along with differences in proportions (Δ) and their corresponding 95% CIs. Between-group comparisons were conducted using Pearson’s chi-square test

Overall, entire intervention period; 1st half, the first half of the intervention period; 2nd half, the second half of the intervention period

*PPS* per protocol set. *P < 0.05, **P < 0.01, ***P < 0.001

During the overall intervention period, common cold symptoms were reported in 73.6% of the participants in the *Euglena* group and 94.4% in the placebo group. The estimated between-group difference was − 20.8% (95% CI: −31.5 to − 10.1; *P* < 0.001), indicating a lower incidence in the *Euglena* group.

During the first half of the intervention, the incidence was 69.2% in the *Euglena* group and 91.1% in the placebo group, corresponding to an estimated between-group difference of − 21.9% (95% CI: −33.5 to − 10.2; *P* < 0.001). During the second half, the incidence rates were 68.1% and 85.6%, respectively, with an estimated between-group difference of − 17.4% (95% CI: −29.7 to − 5.1; *P* = 0.005).

For individual symptoms, lower incidences were observed in the *Euglena* group than in the placebo group for fatigue, chills, runny nose, nasal congestion, sore throat, coughing, and headache during the overall intervention period and the first half of the intervention. In the second half, lower incidences were observed for fatigue, sneezing, chills, and overall common cold symptoms.

No statistically significant between-group differences were observed for other symptoms.

### Bowel movement survey

Bowel movement frequency, number of bowel movement days, and stool volume are presented in Table 4, and stool form is shown in Supplementary Table 6.

**Table 4:** Bowel movement frequency and stool volume during the study period (PPS)

Item	Umit	Time point	*Euglena group*			Placebo group			Group difference			
			*n*	Mean	SD	*n*	Mean	SD	Δ	95%CIs-	95%CIs+	*P* value
Number of bowel movements	day	Period 0	91	5.5	1.8	90	5.2	1.7	0.3	−0.2	0.8	0.205
		Period 1	91	5.7	1.6	90	5.3	1.7	0.2	−0.2	0.5	0.358
		Period 2	91	5.6	1.5	90	5.3	1.7	0.1	−0.2	0.4	0.572
		Period 3	91	5.7	1.6	90	5.4	1.6	0.1	−0.3	0.5	0.572
		Period 4	91	5.8	1.4	90	5.4	1.6	0.3	−0.1	0.6	0.147
		Period 5	91	5.7	1.6	90	5.3	1.7	0.2	−0.2	0.5	0.398
		Period 6	91	5.5	1.6	90	5.4	1.8	−0.1	−0.5	0.3	0.732
		Period 7	91	5.7	1.4	90	5.4	1.8	0.1	−0.2	0.5	0.452
		Period 8	91	5.6	1.4	90	5.2	1.9	0.2	−0.1	0.6	0.215
Frequency of bowel movements	times	Period 0	91	6.6	3.2	90	6.3	3.3	0.3	−0.7	1.2	0.562
		Period 1	91	7.3	3.3	90	6.9	3.7	0.1	−0.5	0.8	0.688
		Period 2	91	7.3	3.2	90	6.8	3.5	0.2	−0.4	0.9	0.495
		Period 3	91	7.3	3.1	90	6.9	3.8	0.2	−0.6	0.9	0.676
		Period 4	91	7.2	3.0	90	6.6	3.1	0.4	−0.3	1.1	0.240
		Period 5	91	7.0	2.9	90	6.7	3.5	0.1	−0.6	0.7	0.812
		Period 6	91	7.0	3.4	90	6.6	3.2	0.1	−0.6	0.8	0.749
		Period 7	91	7.1	3.0	90	6.8	3.5	0.2	−0.5	0.9	0.609
		Period 8	91	6.9	3.0	90	6.5	3.6	0.3	−0.4	0.9	0.464
Amount of stool	container	Period 0	91	35.6	33.6	90	36.3	53.8	−0.7	−13.9	12.5	0.911
		Period 1	91	36.6	35.4	90	32.5	35.8	4.5	−2.8	11.9	0.223
		Period 2	91	39.5	38.4	90	34.6	39.4	5.3	−2.3	13.0	0.171
		Period 3	91	41.3	39.6	90	34.6	34.5	7.2	−0.7	15.1	0.075
		Period 4	91	43.9	46.0	90	34.0	32.6	10.3	0.8	19.8	0.034*
		Period 5	91	42.7	45.1	90	35.6	35.2	7.5	−2.0	17.1	0.122
		Period 6	91	44.1	47.7	90	36.8	37.3	7.7	−2.6	18.0	0.140
		Period 7	91	45.8	51.8	90	38.0	38.0	8.2	−2.9	19.3	0.145
		Period 8	91	46.3	53.0	90	36.8	38.4	10.0	−1.2	21.1	0.080

Data are summarized as the mean and standard deviation (SD). Between-group differences (Δ) at screening are expressed as differences in means. Post-intervention differences are derived from linear mixed models and are presented as differences in estimated marginal means with their corresponding 95% confidence intervals (CIs)

*PPS* per protocol set, *Period 0* 1 week preceding screening, *Period 1–8* each subsequent week from the start to the end of the intervention

*P < 0.05

From intervention days 22 to 28 (period 4), the stool volumes were 43.9 ± 46.0 containers and 34.0 ± 32.6 containers (mean ± SD) in the *Euglena* and placebo groups, respectively. The estimated between-group difference was 10.3 containers (95% CI: 0.8–19.8; *P* = 0.034), indicating a higher stool volume in the *Euglena* group. Throughout the trial period, no statistically significant between-group differences were observed in the *Euglena* group compared with the placebo group.

Regarding the stool form, one participant in the *Euglena* and seven in the placebo groups were classified as “8. Fragmented or rabbit-like” during days 1–7 (adjusted standardized residual: −2.2, *P* = 0.029). Similarly, one and seven participants in the *Euglena* and placebo groups, respectively, were classified as “6. Cylindrical with surface fissures” during days 29–35 (adjusted standardized residual: −2.1, *P* = 0.029). In both cases, fewer participants in the *Euglena* group exhibited these stool forms (Supplementary Table 6).

### Safety analysis

For variables with observed between-group differences, a greater number of participants in the *Euglena* group showed shifts in mean corpuscular volume outside the reference range following the intervention. In contrast, for other variables with between-group differences, higher values were observed in the placebo group. No AEs were reported during the intervention, and no clinically meaningful changes were observed in hematological or urinalysis parameters (Supplementary Tables 7 and 8).

## Discussion

This randomized, double-blind, placebo-controlled trial evaluated the effects of daily consumption of *E. gracilis* as a food-based intervention on immune function and common cold-like symptoms in healthy adults.

The log-transformed CD86 MFI on pDCs at Week 8, which is the primary outcome of this study, was higher in the *Euglena* group than in the placebo group. pDCs act as upstream regulators of immune responses and play pivotal roles in host defense against bacterial and viral infections by contributing to the initiation of inflammation and T-cell immunity in vivo [35]. CD86 is a costimulatory molecule involved in antigen presentation and T-cell activation. Therefore, high CD86 expression on pDCs suggests an enhanced capacity of these cells to participate in immune surveillance and bridge innate and adaptive immune responses. In the context of common cold-like symptoms, this finding may reflect enhanced immune readiness. However, as viral infection, cytokine responses, and downstream T-cell activation were not directly measured, the clinical value of this biomarker should be cautiously interpreted. In addition, among the secondary hematological outcomes, the monocyte percentage was higher in the *Euglena* group at Week 8.

As a prespecified exploratory secondary outcome, the common cold symptom survey showed that, during multiple postintervention periods, fewer participants in the *Euglena* group reported common cold symptoms, including chills, fatigue, sneezing, nasal discharge, nasal congestion, sore throat, cough, and headache. These findings are consistent with previous clinical observations [11–13], although the magnitude and consistency of these differences should be interpreted with caution given the exploratory nature of the endpoint.

Although a direct causal link between pDC activation and symptom improvement cannot be established based on the present results, the overall findings suggest that *E. gracilis* consumption may be associated with the modulation of innate immune responsiveness, as reflected by the elevated expression of CD86 on pDCs. Several potential mechanisms may therefore be considered to explain this observation.

First, paramylon may influence pDC activation via pattern-recognition pathways. DCs serve as key antigen-presenting cells bridging innate and adaptive immunity [36]. Major APC populations include DC subsets, such as pDCs and monocyte-derived cells [37]. Paramylon, the main bioactive component of *E. gracilis*, is a β-1,3-glucan that acts as a pathogen-associated molecular pattern [38]. It binds to Dectin-1, a pattern-recognition receptor and β-glucan receptor expressed on DCs, monocytes, and macrophages, thereby activating the transcription of nuclear factor-kappa B (NF-κB) and inducing innate immune responses in vitro [39–41].

Human pDCs isolated from blood, tonsils, and spleen have also been reported to express Dectin-1 [42]. As paramylon is known to bind Dectin-1 [21, 22, 25], the observed increase in the pDC activation marker CD86 in the present study may indicate stimulation via the Dectin-1 pathway. The activation of Dectin-1 on pDCs promotes downstream signaling via Syk phosphorylation, subsequently inducing the mitogen-activated protein kinase (p38, ERK) and NF-κB pathways, thereby leading to pDC maturation [42]. Alternatively, pDC activation may indirectly occur through cytokines produced by other immune cell populations [43].

A related possibility is that SIRT1-mediated pathways also play a role in paramylon-associated immune regulation. SIRT1 has been implicated in immune regulation, autoimmunity, and immune cell metabolism [44, 45], and has been shown to regulate DC activation during respiratory virus–induced immune responses [46].Furthermore, paramylon extracted from *E. gracilis* EOD-1 has been reported to increase SIRT1 expression [47]. However, plasma SIRT1 levels and SIRT1-related signaling markers were not measured in the present study. Therefore, whether SIRT1 contributed to the changes in pDC CD86 expression or common cold symptoms remains unclear and should be investigated in future studies.

Second, CD86 is a key costimulatory molecule that binds to CD28 on T cells, markedly lowering the activation threshold for antigen presentation by DCs [48, 49]. Supporting this mechanism, the ingestion of *E. gracilis* enriched in paramylon has been shown to promote the activation of naive T cells [12].

Furthermore, monocytes act as precursors that differentiate into macrophages or DCs within peripheral tissues [50]. β-glucans, such as paramylon, have been reported to affect monocyte and macrophage lineage cells [12, 27, 28]. In mice, intraperitoneal administration of *E. gracilis* or paramylon stimulated DCs in Peyer’s patches and modulated immune function [20]. In the present study, we also observed a considerable increase in the percentage of circulating monocytes after *E. gracilis* intake.

Collectively, prior experimental evidence and current human findings suggest that *E. gracilis* consumption may be associated with pDC activation and an increased percentage of precursor cells within the innate immune compartment. As pDCs and monocyte-derived cells constitute major APC populations [37], these changes may reflect coordinated changes in APC-related cell populations, which could be consistent with enhanced immune responsiveness and more efficient initiation of immune responses following exposure to viruses causing common cold symptoms or other pathogens.

Several previous studies have also reported the effects of *Euglena* on colds [11–13]. Although the target populations differed, intake with *E. gracilis* rich in β-1,3-glucan has been reported to reduce the incidence of upper respiratory tract infections and the number of sick days in endurance-trained Western Europeans aged 21–65 years (with a subset of Eastern European and South Asian participants) [11]. Furthermore, studies involving older adults have reported improvements in cold-related symptoms after *E. gracilis* consumption [12]. The present findings are broadly consistent with previous reports suggesting that *E. gracilis* consumption may reduce the cumulative days and severity of common cold symptoms—such as nasal congestion, sore throat, and fatigue—in healthy adults [13]. Collectively, existing evidence supports the potential of *E. gracilis* to maintain and enhance healthy immune function.

In contrast, no statistically significant intergroup difference was observed in the log-transformed MFI of HLA-DR on pDCs at Week 8. During typical pDC activation, MHC class II molecules, such as HLA-DR, are often upregulated alongside costimulatory molecules, such as CD86, allowing pDCs to function as APCs [51]. The asymmetric changes in CD86 and HLA-DR observed in the present study suggest that the primary effect was functional activation of existing pDCs, rather than a substantial increase in pDC numbers or a broad upregulation of all activation markers. Alternatively, stimulation by *E. gracilis* may selectively occur on specific pDC subsets, engaging pathways distinct from those of conventional full maturation. pDCs are widely known to comprise functionally distinct subsets. Conventional pDCs are characterized by their capacity for rapid and robust type I IFN production, whereas CD5⁺ CD81⁺ pDCs demonstrate limited type I IFN secretion but strong capacities for B-cell activation, antibody production, and T-cell proliferation [52]. These subsets are thought to specialize in regulating adaptive immune responses while limiting excessive systemic type I IFN release. Previous experimental evidence has indicated that *Euglena* intake enhances T-cell activation [12] and mucosal immune responses such as increased salivary IgA secretion [14]. Therefore, *E. gracilis* consumption may exert an immunomodulatory effect that selectively enhances pDC-mediated bridging to adaptive immunity, while avoiding a considerable enhancement of systemic immune activation in healthy individuals.

Although *E. gracilis* has been reported to enhance bowel movements [32], the baseline values for bowel movement frequency might explain why no statistically significant intergroup differences were observed in bowel movement frequency or in the number of bowel movement days. According to the Rome IV criteria [53], functional constipation is defined as “fewer than three spontaneous bowel movements per week.” An Internet survey of 5,155 Japanese adults aged 20–79 years (2,542 men and 2,613 women) reported a mean weekly bowel movement frequency of 6.9 ± 4.2 [54].

In the present study, the baseline mean ± SD bowel movement frequencies were 6.6 ± 3.2 and 6.3 ± 3.3 in the *Euglena* and placebo groups, respectively, which are close to the national average. Therefore, potential effects on bowel movement frequency or the number of bowel movement days may have been undetectable.

However, the *Euglena* group showed a higher number of bowel movements during the postintervention period as well as somewhat greater stool volume during specific intervals. In addition, the proportion of participants with stool forms deviating from the optimal “Type 5: smooth, sausage-shaped” category was lower in the *Euglena* group. These findings indicate that *E. gracilis* consumption may exert a modest beneficial influence on bowel function.

Bowel movement outcomes were included as exploratory gastrointestinal measures as *E. gracilis* contains non-digestible components, including paramylon. Although bowel function is potentially relevant to immune regulation through gut–immune interactions, this study did not evaluate gut microbiota, short-chain fatty acids, intestinal permeability, or mucosal immune markers. Therefore, it remains unclear whether the observed bowel-related changes contributed to systemic immune markers or common cold symptoms.

Despite these positive findings, the study has several limitations that need to be acknowledged. Although changes in DC surface markers were evaluated, this study did not directly examine how DC activation influenced the differentiation of T-cell subsets (e.g., Th1, Th2, and Th17), nor did it measure the dynamics of pro- and anti-inflammatory cytokines, such as IL-10, IL-12, or interferons. PBMC processing, staining, flow cytometric acquisition, and gating procedures were standardized, and samples from the *Euglena* and placebo groups were compared under the same analytical conditions to minimize assay-related systematic variability. However, pDC subset composition was not quantified, and subset-specific CD86 expression was not assessed in this study. Therefore, although the observed increase in CD86 MFI may reflect enhanced activation of individual pDCs, we cannot distinguish this from a possible change in the proportion of pDC subsets with higher CD86 expression. Furthermore, blood samples were collected based on the participants’ availability to reflect typical daily living conditions, and the timing of blood collection was not standardized. Therefore, potential diurnal variations in immune parameters cannot be ruled out. In addition, the study population consisted of healthy Japanese adults who were able to attend all scheduled visits at a single clinic in Tokyo. Therefore, the findings may not be generalizable to populations with varying dietary habits, genetic backgrounds, age distributions, health conditions, or baseline immune profiles.

The mechanistic interpretation presented herein is based on indirect and associative evidence rather than direct causal validation. Therefore, the proposed involvement of paramylon-mediated pathways, including Dectin-1 signaling and downstream immune activation, is based on associative evidence and should not be interpreted as establishing causal mechanisms. Moreover, secondary outcomes were prespecified as exploratory, and no formal adjustment for multiplicity was made. Accordingly, these findings should be interpreted with appropriate caution.

Data on daily symptoms were based on participant-reported diaries and were not confirmed by physician diagnosis or virological testing. Therefore, these outcomes are inherently subjective and may be influenced by reporting bias, recall bias, or individual differences in symptom perception. In addition, dietary intake beyond the intervention was not strictly controlled, which may influence the interpretation of the findings within a broader nutritional context. Furthermore, although accumulated evidence suggests that paramylon is likely to be the main contributor to the observed effects, the possibility that other nutritional components of *E. gracilis*, such as amino acids, lipids, and micronutrients, also have contributions cannot be ruled out.

Future research incorporating standardized sampling conditions, detailed pDC subset phenotyping, analysis of other immune cells, simultaneous measurement of cytokines in blood and other compartments, trials using purified paramylon, and studies in more diverse populations will be crucial for elucidating causal pathways, better delineating the immunomodulatory effects of *E. gracilis*, and evaluating the generalizability of these findings.

## Conclusion

Daily intake of *E. gracilis* (1,000 mg/day) for 8 weeks was associated with higher CD86 expression on pDCs and a lower participant-reported incidence of common cold-like symptoms in healthy Japanese adults. These findings suggest that *E. gracilis* may modulate innate immune responses. The observed reduction in common cold symptoms may reflect improved host responsiveness, although causal relationships cannot be established. These results support the potential of *E. gracilis* as a dietary approach for immune health and highlight microalgae as sustainable sources of functional dietary components.

## Supplementary Information

Supplementary Material 1.

## Data Availability

No additional datasets were generated or analysed during the current study.

## Abbreviations

AEs: Adverse events
APCs: Antigen-presenting cells
BMI: Body mass index
CI: Confidence interval
DCs: Dendritic cells
E. gracilis: Euglena gracilis
FFC: Foods with Function Claims
FOSHU: Foods for Specified Health Uses
HLA: Human leukocyte antigen
IFN: Interferon
IgE: Immunoglobulin E
IL: Interleukin
MFI: Median fluorescence intensity
MHC: Major histocompatibility complex
NF-κB: Nuclear factor-kappa B
NK: Natural killer
pDCs: Plasmacytoid dendritic cells
SD: Standard deviation
