# Supplementary material for: Incorporating Euglena gracilis into the habitual diet modulates immune function and reduces common cold-like symptoms: a randomized controlled trial
Source: Nutr J. 2026 Jun 9;25:90. doi: 10.1186/s12937-026-01350-6 (PMC13483718; doi:10.1186/s12937-026-01350-6)
Supplement: Supplementary file 1 [file 12937_2026_1350_MOESM1_ESM.docx]

**Supplementary Material**

**Eligibility Criteria for Study Participants**

Participants who met any of the following conditions were excluded from the study: (1) undergoing medical treatment or having a history of malignant tumor, heart failure, or myocardial infarction; (2) having a pacemaker or an implantable cardioverter defibrillator (ICD); (3) currently receiving treatment for any of the following chronic diseases: cardiac arrhythmia, liver disorder, chronic kidney disease, cerebrovascular disorder, rheumatic disease, diabetes mellitus, dyslipidemia, hypertension, and anemia; (4) having an autoimmune disease; (5) taking “Foods for Specified Health Uses” (FOSHU) or “Foods with Functional Claims” (FFC); (6) regularly taking or using medications (including herbal medicines) or supplements, particularly warfarin, medicines for rhinitis, or antihistamines; (7) using immunosuppressants such as steroids; (8) having known allergies to medicines or foods; (9) pregnant, lactating, or planning to become pregnant during the study; (10) having pollen allergy or perennial allergic rhinitis; (11) currently smoking or have quit smoking within the year before providing consent; (12) excessively consuming alcohol; (13) currently on a restrictive diet; (14) engaging in intense exercise; (15) maintaining an irregular lifestyle due to shift work or other factors; (16) having received vaccinations for infectious disease prevention within 3 months prior to providing consent or scheduled to receive one during the study; (17) enrollment in other clinical studies within 28 days prior to consent or planning to participate in another study during the current trial; or (18) ineligible to participate in the study, as judged by the physician.

**Supplemental Methods**

**Flow cytometric analysis of CD86 Expression on pDCs**

Peripheral blood samples were collected in heparinized tubes after at least 6 h of fasting (water permitted), with participants in a seated position. Blood collection was scheduled according to participant availability. Samples were transported to the contracted analytical laboratory on the same day. On the following day, peripheral blood mononuclear cells (PBMCs) were isolated via centrifugation, washed, counted, and cryopreserved at −80 °C in a cell freezing medium. Subsequently, they were stored in liquid nitrogen within 1–2 days until analysis.

Dendritic cell subsets were identified using the lineage depletion strategy described by Olweus et al. [34]. After thawing, PBMCs were incubated with fluorochrome-conjugated antibody cocktails or corresponding isotype control antibodies for 20 min at 4 °C in the dark. Then, they were washed and analyzed using a Navios EX flow cytometer (Beckman Coulter).

Dead cells were excluded using the 7-AAD viability staining solution (BioLegend, 420403). Lineage-negative cells were identified using FITC-conjugated anti-human lineage cocktail (CD3, CD14, CD19, CD20, and CD56; BioLegend, 348701). HLA-DR-positive cells were detected using the APC/cyanine7 anti-human HLA-DR antibody (BioLegend, 307618). Dendritic cell subsets were defined based on CD11c and CD123 expressions using PE/cyanine7 anti-human CD11c (BioLegend, 301608) and PerCP/cyanine5.5 anti-human CD123 (BioLegend, 306016). Plasmacytoid dendritic cells (pDCs) were defined via sequential gating as Lineage− HLA-DR+ CD11c− CD123+ cells [35].

CD86 expression was evaluated using the PE antihuman CD86 antibody (BioLegend, 305406). For the isotype control setting, a portion of thawed PBMCs from each measurement day was pooled and stained with isotype control antibodies. Positive gates were established via isotype control staining, with approximately 1% positivity used as the threshold.

Flow cytometric data were analyzed using the Kaluza software (Beckman Coulter). The primary endpoint was defined as the log-transformed median fluorescence intensity (MFI) of CD86 within the gated pDC population. All samples were acquired and analyzed in identical instrument settings at a contracted analytical facility following standardized operating procedures.

**Supplementary Table 1:** Trial Schedule

|  | | Briefing | Scr−1week | Scr | Enrollment | Allocation | Post-randomization | | |
| --- | --- | --- | --- | --- | --- | --- | --- | --- | --- |
|  |  |  |  |  |  |  | Start intake | Week 4 | Week 8 |
| Registration | Eligibility screen | X |  |  | X |  |  |  |  |
|  | Informed consent | X |  |  |  |  |  |  |  |
|  | Other procedures | X |  |  |  |  |  |  |  |
|  | Allocation |  |  |  |  | X |  |  |  |
| Intervention | *Euglena* |  |  |  |  |  | ● | ● | ● |
|  | Placebo |  |  |  |  |  | ● | ● | ● |
| Assessments | Immune Indices |  |  | X |  |  |  | X | X |
|  | Common Cold Symptom Survey |  | ● | ● |  |  | ● | ● | ● |
|  | Bowel Movement Survey |  | ● | ● |  |  | ● | ● | ● |
|  | Physical measurements |  |  | X |  |  |  | X | X |
|  | Urinalysis |  |  | X |  |  |  | X | X |
|  | Peripheral blood test |  |  | X |  |  |  | X | X |
|  | Medical interview |  |  | X |  |  |  | X | X |
|  | Dietary survey |  |  | X |  |  |  | X | X |
|  | Diary |  |  |  |  |  | ● | ● | ● |

“X” indicates the item of implementation. “●” shows consecutive daily measurements or intake.

Scr, Screening test; Week 4, 4 weeks after intervention; Week 8, 8 weeks after intervention; Scr−1 week, 1 week before the screening test.

**Supplementary Table 2:** Characteristics of the Study Participants (Physical Characteristics)

| Item | Unit |  | ITT | |  | PPS | |  | SAF | |
| --- | --- | --- | --- | --- | --- | --- | --- | --- | --- | --- |
|  |  |  | *Euglena* group (*n* = 100) | Placebo group (*n* = 100) |  | *Euglena* group (*n* = 91) | Placebo group (*n* = 90) |  | *Euglena* group (*n* = 96) | Placebo group (*n* = 97) |
|  |  |  |  |  |  |  |  |  |  |  |
| Sex | - | Male | 45 (45.0%) | 45 (45.0%) |  | 39 (42.9%) | 42 (46.7%) |  | 43 (44.8%) | 45 (46.4%) |
|  |  | Female | 55 (55.0%) | 55 (55.0%) |  | 52 (57.1%) | 48 (53.3%) |  | 53 (55.2%) | 52 (53.6%) |
| Age | years | Mean (SD) | 49.4 (12.2) | 49.6 (12.1) |  | 50.1 (11.5) | 50.2 (12.2) |  | 49.6 (11.8) | 50.1 (11.9) |
|  |  | P value | 0.907 | |  | 0.944 | |  | 0.761 | |
| Height | cm | Mean (SD) | 164.3 (8.1) | 163.8 (8.3) |  | 163.8 (8.3) | 164.0 (8.5) |  | 164.0 (8.2) | 164.0 (8.4) |
|  |  | P value | 0.701 | |  | 0.887 | |  | 0.938 | |
| Weight | kg | Mean (SD) | 62.3 (14.2) | 61.1 (13.7) |  | 61.6 (13.6) | 61.0 (13.6) |  | 62.1 (14.3) | 61.2 (13.8) |
|  |  | P value | 0.526 | |  | 0.762 | |  | 0.671 | |
| BMI | kg/m^2^ | Mean (SD) | 22.9 (4.1) | 22.6 (3.5) |  | 22.8 (3.9) | 22.4 (3.4) |  | 22.9 (4.2) | 22.6 (3.5) |
|  |  | P value | 0.469 | |  | 0.540 | |  | 0.535 | |
| Systolic blood pressure | mmHg | Mean (SD) | 116.4 (17.3) | 118.6 (19.1) |  | 115.9 (17.4) | 119.2 (19.4) |  | 116.2 (17.4) | 119.1 (19.1) |
|  |  | P value | 0.382 | |  | 0.223 | |  | 0.269 | |
| Diastolic blood pressure | mmHg | Mean (SD) | 75.4 (12.9) | 77.4 (12.9) |  | 75.4 (12.8) | 77.6 (12.9) |  | 75.4 (13.0) | 77.8 (12.8) |
|  |  | P value | 0.264 | |  | 0.242 | |  | 0.187 | |

Sex was summarized as the number and percentage of participants, while all other variables were summarized using the mean and standard deviation (SD).

BMI, body mass index; ITT, intention to treat; PPS, per protocol set; SAF, safety analysis population.

**Supplementary Table 3:** Characteristics of the Study Participants (Immune Function)

| Item | Unit |  | ITT | |  | PPS | |  | SAF | |
| --- | --- | --- | --- | --- | --- | --- | --- | --- | --- | --- |
|  |  |  | *Euglena* group (*n* = 100) | Placebo group (*n* = 100) |  | *Euglena* group (*n* = 91) | Placebo group (*n* = 90) |  | *Euglena* group (*n* = 96) | Placebo group (*n* = 97) |
|  |  |  |  |  |  |  |  |  |  |  |
| pDC CD86 MFI | - | Mean (SD) | 3491.4 (1560.9) | 3522.5 (1610.7) |  | 3501.6 (1538.2) | 3522.5 (1589.2) |  | 3523.1 (1561.7) | 3544.2 (1618.5) |
|  |  | P value | 0.890 | |  | 0.928 | |  | 0.927 | |
| pDC HLR-DR MFI | - | Mean (SD) | 141042.3 (48158.6) | 143338.8 (44911.4) |  | 141404.7 (48159.9) | 143150.6 (46118.1) |  | 140229.0 (48835.9) | 143223.3 (45176.7) |
|  |  | P value | 0.728 | |  | 0.804 | |  | 0.659 | |
| Presence/absence of common cold symptoms in the past two years | - | Presence | 100 (100.0%) | 100 (100.0%) |  | 91 (100.0%) | 90 (100.0%) |  | 96 (100.0%) | 97 (100.0%) |
|  |  | Absence | 0 (0.0%) | 0 (0.0%) |  | 0 (0.0%) | 0 (0.0%) |  | 0 (0.0%) | 0 (0.0%) |
|  |  | P value | NA | |  | NA | |  | NA | |
| Presence/absence of common cold symptoms during the week prior to screening | - | Presence | 70 (70.0%) | 67 (67.0%) |  | 62 (68.1%) | 60 (66.7%) |  | 66 (68.8%) | 64 (66.0%) |
|  |  | Absence | 30 (30.0%) | 33 (33.0%) |  | 29 (31.9%) | 30 (33.3%) |  | 30 (31.3%) | 33 (34.0%) |
|  |  | P value | 0.648 | |  | 0.833 | |  | 0.681 | |
| Cedar-specific IgE | UA/mL | Mean (SD) | 5.2 (8.9) | 5.4 (8.7) |  | 5.0 (8.0) | 5.4 (8.9) |  | 4.7 (7.9) | 5.2 (8.7) |
|  |  | P value | 0.890 | |  | 0.750 | |  | 0.681 | |
| Cypress-specific IgE | UA/mL | Mean (SD) | 1.1 (2.2) | 1.0 (1.6) |  | 1.0 (2.2) | 0.9 (1.6) |  | 1.0 (2.1) | 0.9 (1.5) |
|  |  | P value | 0.782 | |  | 0.763 | |  | 0.825 | |

Symptoms of common cold were summarized as the number and percentage of participants, while all other variables were summarized using the mean and standard deviation (SD).

pDC, plasmacytoid dendritic cell; MFI, median fluorescence intensity; HLA, human leukocyte antigen; IgE, immunoglobulin E; ITT, intention to treat; PPS, per protocol set; SAF, safety analysis population; NA, Not Available.

**Supplementary Table 4:** Characteristics of the Study Participants (Bowel Movement Status)

| Item | Unit |  | ITT | |  | PPS | |  | SAF | |
| --- | --- | --- | --- | --- | --- | --- | --- | --- | --- | --- |
|  |  |  | *Euglena* group (*n* = 100) | Placebo group (*n* = 100) |  | *Euglena* group (*n* = 91) | Placebo group (*n* = 90) |  | *Euglena* group (*n* = 96) | Placebo group (*n* = 97) |
|  |  |  |  |  |  |  |  |  |  |  |
| Number of bowel movements in the week prior to screening | day | Mean (SD) | 5.5 (1.8) | 5.2 (1.7) |  | 5.5 (1.8) | 5.2 (1.7) |  | 5.6 (1.8) | 5.2 (1.6) |
|  |  | P value | 0.171 | |  | 0.205 | |  | 0.188 | |
| Frequency of bowel movements in the week prior to screening | times | Mean (SD) | 6.7 (3.2) | 6.3 (3.3) |  | 6.6 (3.2) | 6.3 (3.3) |  | 6.7 (3.2) | 6.3 (3.3) |
|  |  | P value | 0.399 | |  | 0.562 | |  | 0.433 | |
| Amount of stool in the week prior to screening | container | Mean (SD) | 39.5 (40.5) | 35.3 (52.3) |  | 35.6 (33.6) | 36.3 (53.8) |  | 38.8 (39.7) | 36.0 (52.9) |
|  |  | P value | 0.520 | |  | 0.911 | |  | 0.687 | |
| Numerical response for stool form in the week prior to screening | - | Mean (SD) | 4.9 (1.0) | 5.0 (1.2) |  | 5.0 (1.0) | 5.0 (1.2) |  | 5.0 (1.0) | 5.0 (1.2) |
|  |  | P value | 0.514 | |  | 0.946 | |  | 0.786 | |

Data were summarized using the mean and standard deviation (SD).

ITT, intention to treat; PPS, per protocol set; SAF, safety analysis population.

**Supplementary Table 5:** Characteristics of Study Participants (Presence/absence of common cold symptoms)

| Item | Unit |  | ITT | |  | PPS | |  | SAF | |
| --- | --- | --- | --- | --- | --- | --- | --- | --- | --- | --- |
|  |  |  | *Euglena* group (*n* = 100) | Placebo group (*n* = 100) |  | *Euglena* group (*n* = 91) | Placebo group (*n* = 90) |  | *Euglena* group (*n* = 96) | Placebo group (*n* = 97) |
|  |  |  |  |  |  |  |  |  |  |  |
| Presence/absence of general malaise in the past two years | - | Presence | 28 (28.0%) | 26 (26.0%) |  | 25 (27.5%) | 25 (27.8%) |  | 27 (28.1%) | 26 (26.8%) |
|  |  | Absence | 72 (72.0%) | 74 (74.0%) |  | 66 (72.5%) | 65 (72.2%) |  | 69 (71.9%) | 71 (73.2%) |
|  |  | P value | 0.750 | |  | 0.963 | |  | 0.837 | |
| Presence/absence of chills in the past two years | - | Presence | 9 (9.0%) | 14 (14.0%) |  | 7 (7.7%) | 14 (15.6%) |  | 8 (8.3%) | 14 (14.4%) |
|  |  | Absence | 91 (91.0%) | 86 (86.0%) |  | 84 (92.3%) | 76 (84.4%) |  | 88 (91.7%) | 83 (85.6%) |
|  |  | P value | 0.268 | |  | 0.099 | |  | 0.182 | |
| Presence/absence of feverishness in the past two years | - | Presence | 8 (8.0%) | 13 (13.0%) |  | 7 (7.7%) | 13 (14.4%) |  | 7 (7.3%) | 13 (13.4%) |
|  |  | Absence | 92 (92.0%) | 87 (87.0%) |  | 84 (92.3%) | 77 (85.6%) |  | 89 (92.7%) | 84 (86.6%) |
|  |  | P value | 0.249 | |  | 0.147 | |  | 0.164 | |
| Presence/absence of fatigue in the past two years | - | Presence | 37 (37.0%) | 37 (37.0%) |  | 32 (35.2%) | 34 (37.8%) |  | 35 (36.5%) | 37 (38.1%) |
|  |  | Absence | 63 (63.0%) | 63 (63.0%) |  | 59 (64.8%) | 56 (62.2%) |  | 61 (63.5%) | 60 (61.9%) |
|  |  | P value | 1.000 | |  | 0.715 | |  | 0.809 | |
| Presence/absence of sneezing in the past two years | - | Presence | 29 (29.0%) | 30 (30.0%) |  | 27 (29.7%) | 27 (30.0%) |  | 28 (29.2%) | 28 (28.9%) |
|  |  | Absence | 71 (71.0%) | 70 (70.0%) |  | 64 (70.3%) | 63 (70.0%) |  | 68 (70.8%) | 69 (71.1%) |
|  |  | P value | 0.877 | |  | 0.961 | |  | 0.963 | |
| Presence/absence of runny nose in the past two years | - | Presence | 35 (35.0%) | 43 (43.0%) |  | 31 (34.1%) | 38 (42.2%) |  | 34 (35.4%) | 43 (44.3%) |
|  |  | Absence | 65 (65.0%) | 57 (57.0%) |  | 60 (65.9%) | 52 (57.8%) |  | 62 (64.6%) | 54 (55.7%) |
|  |  | P value | 0.246 | |  | 0.259 | |  | 0.206 | |
| Presence/absence of nasal congestion in the past two years | - | Presence | 19 (19.0%) | 31 (31.0%) |  | 17 (18.7%) | 27 (30.0%) |  | 18 (18.8%) | 30 (30.9%) |
|  |  | Absence | 81 (81.0%) | 69 (69.0%) |  | 74 (81.3%) | 63 (70.0%) |  | 78 (81.3%) | 67 (69.1%) |
|  |  | P value | 0.050 | |  | 0.076 | |  | 0.050 | |
| Presence/absence of sore throat in the past two years | - | Presence | 22 (22.0%) | 26 (26.0%) |  | 21 (23.1%) | 24 (26.7%) |  | 21 (21.9%) | 26 (26.8%) |
|  |  | Absence | 78 (78.0%) | 74 (74.0%) |  | 70 (76.9%) | 66 (73.3%) |  | 75 (78.1%) | 71 (73.2%) |
|  |  | P value | 0.508 | |  | 0.576 | |  | 0.425 | |
| Presence/absence of coughing in the past two years | - | Presence | 22 (22.0%) | 31 (31.0%) |  | 20 (22.0%) | 31 (34.4%) |  | 20 (20.8%) | 31 (32.0%) |
|  |  | Absence | 78 (78.0%) | 69 (69.0%) |  | 71 (78.0%) | 59 (65.6%) |  | 76 (79.2%) | 66 (68.0%) |
|  |  | P value | 0.149 | |  | 0.062 | |  | 0.080 | |

**Supplementary Table 5:** Characteristics of Study Participants (Presence/absence of common cold symptoms) (continued)

| Item | Unit |  | ITT | |  | PPS | |  | SAF | |
| --- | --- | --- | --- | --- | --- | --- | --- | --- | --- | --- |
|  |  |  | *Euglena* group (*n* = 100) | Placebo group (*n* = 100) |  | *Euglena* group (*n* = 91) | Placebo group (*n* = 90) |  | *Euglena* group (*n* = 96) | Placebo group (*n* = 97) |
|  |  |  |  |  |  |  |  |  |  |  |
| Presence/absence of arthralgia in the past two years | - | Presence | 11 (11.0%) | 14 (14.0%) |  | 9 (9.9%) | 13 (14.4%) |  | 9 (9.4%) | 14 (14.4%) |
|  |  | Absence | 89 (89.0%) | 86 (86.0%) |  | 82 (90.1%) | 77 (85.6%) |  | 87 (90.6%) | 83 (85.6%) |
|  |  | P value | 0.521 | |  | 0.348 | |  | 0.278 | |
| Presence/absence of myalgia in the past two years | - | Presence | 10 (10.0%) | 11 (11.0%) |  | 9 (9.9%) | 11 (12.2%) |  | 9 (9.4%) | 11 (11.3%) |
|  |  | Absence | 90 (90.0%) | 89 (89.0%) |  | 82 (90.1%) | 79 (87.8%) |  | 87 (90.6%) | 86 (88.7%) |
|  |  | P value | 0.818 | |  | 0.617 | |  | 0.654 | |
| Presence/absence of headache in the past two years | - | Presence | 12 (12.0%) | 16 (16.0%) |  | 10 (11.0%) | 15 (16.7%) |  | 11 (11.5%) | 16 (16.5%) |
|  |  | Absence | 88 (88.0%) | 84 (84.0%) |  | 81 (89.0%) | 75 (83.3%) |  | 85 (88.5%) | 81 (83.5%) |
|  |  | P value | 0.415 | |  | 0.268 | |  | 0.313 | |
| Presence/absence of general malaise during the week prior to screening | - | Presence | 36 (36.0%) | 29 (29.0%) |  | 33 (36.3%) | 25 (27.8%) |  | 35 (36.5%) | 28 (28.9%) |
|  |  | Absence | 64 (64.0%) | 71 (71.0%) |  | 58 (63.7%) | 65 (72.2%) |  | 61 (63.5%) | 69 (71.1%) |
|  |  | P value | 0.291 | |  | 0.221 | |  | 0.261 | |
| Presence/absence of chills during the week prior to screening | - | Presence | 11 (11.0%) | 11 (11.0%) |  | 8 (8.8%) | 10 (11.1%) |  | 10 (10.4%) | 11 (11.3%) |
|  |  | Absence | 89 (89.0%) | 89 (89.0%) |  | 83 (91.2%) | 80 (88.9%) |  | 86 (89.6%) | 86 (88.7%) |
|  |  | P value | 1.000 | |  | 0.602 | |  | 0.837 | |
| Presence/absence of feverishness during the week prior to screening | - | Presence | 5 (5.0%) | 7 (7.0%) |  | 5 (5.5%) | 7 (7.8%) |  | 5 (5.2%) | 7 (7.2%) |
|  |  | Absence | 95 (95.0%) | 93 (93.0%) |  | 86 (94.5%) | 83 (92.2%) |  | 91 (94.8%) | 90 (92.8%) |
|  |  | P value | 0.552 | |  | 0.537 | |  | 0.563 | |
| Presence/absence of fatigue during the week prior to screening | - | Presence | 60 (60.0%) | 50 (50.0%) |  | 54 (59.3%) | 43 (47.8%) |  | 57 (59.4%) | 47 (48.5%) |
|  |  | Absence | 40 (40.0%) | 50 (50.0%) |  | 37 (40.7%) | 47 (52.2%) |  | 39 (40.6%) | 50 (51.5%) |
|  |  | P value | 0.155 | |  | 0.119 | |  | 0.128 | |
| Presence/absence of sneezing during the week prior to screening | - | Presence | 19 (19.0%) | 14 (14.0%) |  | 17 (18.7%) | 13 (14.4%) |  | 18 (18.8%) | 14 (14.4%) |
|  |  | Absence | 81 (81.0%) | 86 (86.0%) |  | 74 (81.3%) | 77 (85.6%) |  | 78 (81.3%) | 83 (85.6%) |
|  |  | P value | 0.341 | |  | 0.443 | |  | 0.420 | |
| Presence/absence of runny nose during the week prior to screening | - | Presence | 26 (26.0%) | 26 (26.0%) |  | 23 (25.3%) | 24 (26.7%) |  | 24 (25.0%) | 26 (26.8%) |
|  |  | Absence | 74 (74.0%) | 74 (74.0%) |  | 68 (74.7%) | 66 (73.3%) |  | 72 (75.0%) | 71 (73.2%) |
|  |  | P value | 1.000 | |  | 0.831 | |  | 0.775 | |

**Supplementary Table 5:** Characteristics of Study Participants (Presence/absence of common cold symptoms) (continued)

| Item | Unit |  | ITT | |  | PPS | |  | SAF | |
| --- | --- | --- | --- | --- | --- | --- | --- | --- | --- | --- |
|  |  |  | *Euglena* group (*n* = 100) | Placebo group (*n* = 100) |  | *Euglena* group (*n* = 91) | Placebo group (*n* = 90) |  | *Euglena* group (*n* = 96) | Placebo group (*n* = 97) |
|  |  |  |  |  |  |  |  |  |  |  |
| Presence/absence of nasal congestion during the week prior to screening | - | Presence | 13 (13.0%) | 11 (11.0%) |  | 13 (14.3%) | 11 (12.2%) |  | 13 (13.5%) | 11 (11.3%) |
|  |  | Absence | 87 (87.0%) | 89 (89.0%) |  | 78 (85.7%) | 79 (87.8%) |  | 83 (86.5%) | 86 (88.7%) |
|  |  | P value | 0.663 | |  | 0.682 | |  | 0.643 | |
| Presence/absence of sore throat during the week prior to screening | - | Presence | 18 (18.0%) | 19 (19.0%) |  | 17 (18.7%) | 18 (20.0%) |  | 17 (17.7%) | 19 (19.6%) |
|  |  | Absence | 82 (82.0%) | 81 (81.0%) |  | 74 (81.3%) | 72 (80.0%) |  | 79 (82.3%) | 78 (80.4%) |
|  |  | P value | 0.856 | |  | 0.822 | |  | 0.738 | |
| Presence/absence of coughing during the week prior to screening | - | Presence | 11 (11.0%) | 13 (13.0%) |  | 10 (11.0%) | 13 (14.4%) |  | 10 (10.4%) | 13 (13.4%) |
|  |  | Absence | 89 (89.0%) | 87 (87.0%) |  | 81 (89.0%) | 77 (85.6%) |  | 86 (89.6%) | 84 (86.6%) |
|  |  | P value | 0.663 | |  | 0.485 | |  | 0.522 | |
| Presence/absence of arthralgia during the week prior to screening | - | Presence | 12 (12.0%) | 10 (10.0%) |  | 11 (12.1%) | 7 (7.8%) |  | 12 (12.5%) | 10 (10.3%) |
|  |  | Absence | 88 (88.0%) | 90 (90.0%) |  | 80 (87.9%) | 83 (92.2%) |  | 84 (87.5%) | 87 (89.7%) |
|  |  | P value | 0.651 | |  | 0.333 | |  | 0.632 | |
| Presence/absence of myalgia during the week prior to screening | - | Presence | 17 (17.0%) | 14 (14.0%) |  | 15 (16.5%) | 14 (15.6%) |  | 15 (15.6%) | 14 (14.4%) |
|  |  | Absence | 83 (83.0%) | 86 (86.0%) |  | 76 (83.5%) | 76 (84.4%) |  | 81 (84.4%) | 83 (85.6%) |
|  |  | P value | 0.558 | |  | 0.865 | |  | 0.817 | |
| Presence/absence of headache during the week prior to screening | - | Presence | 16 (16.0%) | 17 (17.0%) |  | 15 (16.5%) | 14 (15.6%) |  | 16 (16.7%) | 17 (17.5%) |
|  |  | Absence | 84 (84.0%) | 83 (83.0%) |  | 76 (83.5%) | 76 (84.4%) |  | 80 (83.3%) | 80 (82.5%) |
|  |  | P value | 0.849 | |  | 0.865 | |  | 0.874 | |

The number and percentage of applicable participants are presented.

**Supplementary Table 6:** Between-group comparison of stool form responses

| Item | Time point | Group | 1. Watery diarrhea and very rapid passage | 2. Mushy, flat surface, somewhat rapid passage | 3. Mushy, uneven surface | 4. Partly broken shape | 5. Cylindrical, smooth surface | 6. Cylindrical, surface cracks | 7. Cylindrical, deep cracks. | 8. Fragmented or pellet-like (similar to deer/rabbit droppings) |
| --- | --- | --- | --- | --- | --- | --- | --- | --- | --- | --- |
| Stool form | Period 0 | Frequency in the *Euglena* group | 0 | 1 | 5 | 16 | 47 | 16 | 4 | 1 |
|  |  | Frequency in the placebo group | 0 | 4 | 2 | 18 | 44 | 16 | 1 | 5 |
|  |  | Adjusted residuals | - | −1.4 | 1.2 | −0.4 | 0.4 | 0.0 | 1.4 | −1.7 |
|  |  | P value | - | 0.174 | 0.247 | 0.703 | 0.655 | 1.000 | 0.174 | 0.097 |
|  | Period 1 | Frequency in the *Euglena* group | 0 | 2 | 4 | 15 | 58 | 7 | 4 | 1 |
|  |  | Frequency in the placebo group | 0 | 2 | 3 | 16 | 53 | 7 | 2 | 7 |
|  |  | Adjusted residuals | - | 0.0 | 0.4 | −0.2 | 0.7 | 0.0 | 0.8 | −2.2 |
|  |  | P value | - | 0.991 | 0.711 | 0.817 | 0.503 | 0.983 | 0.414 | 0.029* |
|  | Period 2 | Frequency in the *Euglena* group | 0 | 2 | 2 | 26 | 48 | 8 | 2 | 3 |
|  |  | Frequency in the placebo group | 0 | 3 | 7 | 17 | 43 | 13 | 1 | 6 |
|  |  | Adjusted residuals | - | −0.5 | −1.7 | 1.5 | 0.7 | −1.2 | 0.6 | −1.0 |
|  |  | P value | - | 0.641 | 0.084 | 0.126 | 0.504 | 0.235 | 0.567 | 0.297 |

**Supplementary Table 6:** Between-group comparison of stool form responses (continued)

| Item | Time point | Group | 1. Watery diarrhea and very rapid passage | 2. Mushy, flat surface, somewhat rapid passage | 3. Mushy, uneven surface | 4. Partly broken shape | 5. Cylindrical, smooth surface | 6. Cylindrical, surface cracks | 7. Cylindrical, deep cracks. | 8. Fragmented or pellet-like (similar to deer/rabbit droppings) |
| --- | --- | --- | --- | --- | --- | --- | --- | --- | --- | --- |
| Stool form | Period 3 | Frequency in the *Euglena* group | 0 | 4 | 4 | 15 | 48 | 13 | 5 | 2 |
|  |  | Frequency in the placebo group | 0 | 2 | 4 | 20 | 49 | 8 | 3 | 4 |
|  |  | Adjusted residuals | - | 0.8 | 0.0 | −1.0 | −0.2 | 1.1 | 0.7 | −0.8 |
|  |  | P value | - | 0.414 | 0.987 | 0.328 | 0.819 | 0.257 | 0.479 | 0.399 |
|  | Period 4 | Frequency in the *Euglena* group | 0 | 2 | 1 | 19 | 52 | 9 | 4 | 4 |
|  |  | Frequency in the placebo group | 0 | 1 | 5 | 15 | 50 | 15 | 2 | 2 |
|  |  | Adjusted residuals | - | 0.6 | −1.7 | 0.7 | 0.2 | −1.3 | 0.8 | 0.8 |
|  |  | P value | - | 0.567 | 0.094 | 0.468 | 0.830 | 0.179 | 0.414 | 0.414 |
|  | Period 5 | Frequency in the *Euglena* group | 0 | 1 | 1 | 24 | 53 | 6 | 2 | 4 |
|  |  | Frequency in the placebo group | 1 | 3 | 4 | 17 | 42 | 15 | 6 | 2 |
|  |  | Adjusted residuals | −1.0 | −1.0 | −1.4 | 1.2 | 1.6 | −2.1 | −1.5 | 0.8 |
|  |  | P value | 0.313 | 0.307 | 0.170 | 0.229 | 0.119 | 0.034* | 0.144 | 0.414 |

**Supplementary Table 6:** Between-group comparison of stool form responses (continued)

| Item | Time point | Group | 1. Watery diarrhea and very rapid passage | 2. Mushy, flat surface, somewhat rapid passage | 3. Mushy, uneven surface | 4. Partly broken shape | 5. Cylindrical, smooth surface | 6. Cylindrical, surface cracks | 7. Cylindrical, deep cracks. | 8. Fragmented or pellet-like (similar to deer/rabbit droppings) |
| --- | --- | --- | --- | --- | --- | --- | --- | --- | --- | --- |
| Stool form | Period 6 | Frequency in the *Euglena* group | 0 | 2 | 4 | 18 | 52 | 9 | 3 | 3 |
|  |  | Frequency in the placebo group | 0 | 1 | 6 | 19 | 44 | 12 | 3 | 4 |
|  |  | Adjusted residuals | - | 0.6 | −0.7 | −0.3 | 1.0 | −0.8 | 0.0 | −0.4 |
|  |  | P value | - | 0.574 | 0.492 | 0.795 | 0.300 | 0.453 | 0.978 | 0.678 |
|  | Period 7 | Frequency in the *Euglena* group | 0 | 2 | 2 | 25 | 49 | 8 | 2 | 3 |
|  |  | Frequency in the placebo group | 0 | 1 | 4 | 23 | 43 | 8 | 6 | 4 |
|  |  | Adjusted residuals | - | 0.6 | −0.9 | 0.2 | 0.7 | 0.0 | −1.5 | −0.4 |
|  |  | P value | - | 0.574 | 0.391 | 0.805 | 0.458 | 0.963 | 0.139 | 0.678 |
|  | Period 8 | Frequency in the *Euglena* group | 0 | 1 | 4 | 20 | 51 | 10 | 2 | 3 |
|  |  | Frequency in the placebo group | 0 | 3 | 4 | 17 | 38 | 19 | 5 | 3 |
|  |  | Adjusted residuals | - | −1.0 | 0.0 | 0.5 | 1.8 | −1.9 | −1.2 | 0.0 |
|  |  | P value | - | 0.301 | 0.974 | 0.633 | 0.073 | 0.059 | 0.235 | 0.978 |

The number of participants corresponding to each response is presented. Between-group differences were assessed using Pearson’s chi-square test.

Period 0, 1 week preceding screening; Period 1–8, each subsequent week from the start to the end of the intervention.

**P* < 0.05

**Supplementary Table 7:** Confirmation of adverse events

| Item | *Euglena* group | | |  | Placebo group | | |  | Group difference | | | | | | |
| --- | --- | --- | --- | --- | --- | --- | --- | --- | --- | --- | --- | --- | --- | --- | --- |
|  | *n* | Number of participants | Number of occurrences  (%) |  | *n* | Number of participants | Number of occurrences  (%) |  | ORs | 95% CIs− | 95% CIs+ | Δ (%) | 95% CIs− | 95% CIs+ | P value |
| Participants who experienced adverse events | 96 | 0 | 0.0 |  | 97 | 0 | 0.0 |  | NA | NA | NA | 0.0 | NA | NA | NA |

The number and proportion of applicable cases are reported, and between-group comparisons are presented as odds ratios (ORs) with 95% confidence intervals (CIs), along with differences in proportions (Δ) and their corresponding 95% CIs. Between-group comparisons were conducted using Pearson’s chi-square test.

NA, Not Available.

**Supplementary Table 8:** Participants showing out-of-range values after intervention (screening values normal)

| Item | Time point | *Euglena* group | | |  | Placebo group | | |  | Group difference | | | | | | |
| --- | --- | --- | --- | --- | --- | --- | --- | --- | --- | --- | --- | --- | --- | --- | --- | --- |
|  |  | *n* | Number of participants | Number of occurrences  (%) |  | *n* | Number of participants | Number of occurrences  (%) |  | ORs | 95% CIs− | 95% CIs+ | Δ (%) | 95% CIs− | 95% CIs+ | P value |
| Urine protein (qualitative) | Week 4 | 95 | 3 | 3.2 |  | 97 | 2 | 2.1 |  | 1.55 | 0.25 | 9.48 | 1.1 | −3.4 | 5.6 | 0.634 |
|  | Week 8 | 95 | 5 | 5.3 |  | 95 | 2 | 2.1 |  | 2.58 | 0.49 | 13.66 | 3.2 | −2.2 | 8.5 | 0.248 |
| Urine glucose (qualitative) | Week 4 | 95 | 1 | 1.1 |  | 97 | 3 | 3.1 |  | 0.33 | 0.03 | 3.26 | −2.0 | −6.1 | 2.0 | 0.322 |
|  | Week 8 | 95 | 1 | 1.1 |  | 95 | 1 | 1.1 |  | 1.00 | 0.06 | 16.22 | 0.0 | −2.9 | 2.9 | 1.000 |
| Urine pH | Week 4 | 95 | 0 | 0.0 |  | 97 | 0 | 0.0 |  | NA | NA | NA | 0.0 | NA | NA | NA |
|  | Week 8 | 95 | 1 | 1.1 |  | 95 | 1 | 1.1 |  | 1.00 | 0.06 | 16.22 | 0.0 | −2.9 | 2.9 | 1.000 |
| Urine occult blood | Week 4 | 95 | 11 | 11.6 |  | 97 | 5 | 5.2 |  | 2.41 | 0.80 | 7.22 | 6.4 | −1.4 | 14.2 | 0.107 |
|  | Week 8 | 95 | 3 | 3.2 |  | 95 | 2 | 2.1 |  | 1.52 | 0.25 | 9.29 | 1.1 | −3.5 | 5.6 | 0.650 |
| White blood cell count | Week 4 | 95 | 6 | 6.3 |  | 97 | 6 | 6.2 |  | 1.02 | 0.32 | 3.29 | 0.1 | −6.7 | 7.0 | 0.970 |
|  | Week 8 | 95 | 4 | 4.2 |  | 95 | 5 | 5.3 |  | 0.79 | 0.21 | 3.04 | −1.1 | −7.1 | 5.0 | 0.733 |
| Red blood cell count | Week 4 | 95 | 6 | 6.3 |  | 97 | 2 | 2.1 |  | 3.20 | 0.63 | 16.28 | 4.3 | −1.4 | 9.9 | 0.140 |
|  | Week 8 | 95 | 7 | 7.4 |  | 95 | 4 | 4.2 |  | 1.81 | 0.51 | 6.40 | 3.2 | −3.5 | 9.8 | 0.351 |
| Hemoglobin | Week 4 | 95 | 2 | 2.1 |  | 97 | 5 | 5.2 |  | 0.40 | 0.07 | 2.09 | −3.0 | −8.4 | 2.3 | 0.260 |
|  | Week 8 | 95 | 4 | 4.2 |  | 95 | 9 | 9.5 |  | 0.42 | 0.12 | 1.41 | −5.3 | −12.4 | 1.9 | 0.151 |
| Hematocrit | Week 4 | 95 | 6 | 6.3 |  | 97 | 3 | 3.1 |  | 2.11 | 0.51 | 8.70 | 3.2 | −2.8 | 9.2 | 0.291 |
|  | Week 8 | 95 | 4 | 4.2 |  | 95 | 5 | 5.3 |  | 0.79 | 0.21 | 3.04 | −1.1 | −7.1 | 5.0 | 0.733 |
| Platelet count | Week 4 | 95 | 3 | 3.2 |  | 97 | 8 | 8.2 |  | 0.36 | 0.09 | 1.41 | −5.1 | −11.7 | 1.5 | 0.129 |
|  | Week 8 | 95 | 2 | 2.1 |  | 95 | 5 | 5.3 |  | 0.39 | 0.07 | 2.05 | −3.2 | −8.5 | 2.2 | 0.248 |
| Mean corpuscular volume | Week 4 | 95 | 4 | 4.2 |  | 97 | 0 | 0.0 |  | NA | NA | NA | 4.2 | 0.2 | 8.3 | 0.041* |
|  | Week 8 | 95 | 3 | 3.2 |  | 95 | 2 | 2.1 |  | 1.52 | 0.25 | 9.29 | 1.1 | −3.5 | 5.6 | 0.650 |
| Mean corpuscular hemoglobin | Week 4 | 95 | 3 | 3.2 |  | 97 | 2 | 2.1 |  | 1.55 | 0.25 | 9.48 | 1.1 | −3.4 | 5.6 | 0.634 |
|  | Week 8 | 95 | 4 | 4.2 |  | 95 | 3 | 3.2 |  | 1.35 | 0.29 | 6.19 | 1.1 | −4.3 | 6.4 | 0.700 |
| Mean corpuscular hemoglobin concentration | Week 4 | 95 | 2 | 2.1 |  | 97 | 3 | 3.1 |  | 0.67 | 0.11 | 4.13 | −1.0 | −5.5 | 3.5 | 0.668 |
|  | Week 8 | 95 | 1 | 1.1 |  | 95 | 5 | 5.3 |  | 0.19 | 0.02 | 1.67 | −4.2 | −9.2 | 0.8 | 0.097 |
| Neutrophil percentage | Week 4 | 95 | 4 | 4.2 |  | 97 | 2 | 2.1 |  | 2.09 | 0.37 | 11.68 | 2.1 | −2.8 | 7.1 | 0.392 |
|  | Week 8 | 95 | 2 | 2.1 |  | 95 | 3 | 3.2 |  | 0.66 | 0.11 | 4.04 | −1.1 | −5.6 | 3.5 | 0.650 |

**Supplementary Table 8:** Participants showing out-of-range values after intervention (screening values normal) (continued)

| Item | Time point | *Euglena* group | | |  | Placebo group | | |  | Group difference | | | | | | | |
| --- | --- | --- | --- | --- | --- | --- | --- | --- | --- | --- | --- | --- | --- | --- | --- | --- | --- |
|  |  | *n* | Number of participants | Number of occurrences  (%) |  | *n* | Number of participants | Number of occurrences  (%) |  | ORs | 95% CIs− | 95% CIs+ | Δ (%) | 95% CIs− | 95% CIs+ | P value | |
| Lymphocyte percentage | Week 4 | 95 | 4 | 4.2 |  | 97 | 1 | 1.0 |  | 4.22 | 0.46 | 38.47 | 3.2 | −1.3 | 7.7 | 0.167 | |
|  | Week 8 | 95 | 3 | 3.2 |  | 95 | 4 | 4.2 |  | 0.74 | 0.16 | 3.41 | −1.1 | −6.4 | 4.3 | 0.700 | |
| Monocyte percentage | Week 4 | 95 | 0 | 0.0 |  | 97 | 0 | 0.0 |  | NA | NA | NA | 0.0 | NA | NA | NA | |
|  | Week 8 | 95 | 2 | 2.1 |  | 95 | 0 | 0.0 |  | NA | NA | NA | 2.1 | −0.8 | 5.0 | 0.155 | |
| Eosinophil percentage | Week 4 | 95 | 5 | 5.3 |  | 97 | 2 | 2.1 |  | 2.64 | 0.50 | 13.95 | 3.2 | −2.1 | 8.5 | 0.237 | |
|  | Week 8 | 95 | 1 | 1.1 |  | 95 | 2 | 2.1 |  | 0.49 | 0.04 | 5.55 | −1.1 | −4.6 | 2.5 | 0.561 | |
| Basophil percentage | Week 4 | 95 | 2 | 2.1 |  | 97 | 2 | 2.1 |  | 1.02 | 0.14 | 7.40 | 0.0 | −4.0 | 4.1 | 0.983 | |
|  | Week 8 | 95 | 1 | 1.1 |  | 95 | 0 | 0.0 |  | NA | NA | NA | 1.1 | −1.0 | 3.1 | 0.316 | |
| Aspartate aminotransferase | Week 4 | 95 | 1 | 1.1 |  | 97 | 0 | 0.0 |  | NA | NA | NA | 1.1 | −1.0 | 3.1 | 0.311 | |
|  | Week 8 | 95 | 2 | 2.1 |  | 95 | 1 | 1.1 |  | 2.02 | 0.18 | 22.68 | 1.1 | −2.5 | 4.6 | 0.561 | |
| Alanine aminotransferase | Week 4 | 95 | 2 | 2.1 |  | 97 | 3 | 3.1 |  | 0.67 | 0.11 | 4.13 | −1.0 | −5.5 | 3.5 | 0.668 | |
|  | Week 8 | 95 | 3 | 3.2 |  | 95 | 1 | 1.1 |  | 3.07 | 0.31 | 30.01 | 2.1 | −2.0 | 6.2 | 0.312 | |
| Gamma-glutamyl transferase | Week 4 | 95 | 3 | 3.2 |  | 97 | 1 | 1.0 |  | 3.13 | 0.32 | 30.64 | 2.1 | −1.9 | 6.2 | 0.302 | |
|  | Week 8 | 95 | 4 | 4.2 |  | 95 | 2 | 2.1 |  | 2.04 | 0.37 | 11.44 | 2.1 | −2.9 | 7.1 | 0.407 | |
| Total bilirubin | Week 4 | 95 | 1 | 1.1 |  | 97 | 1 | 1.0 |  | 1.02 | 0.06 | 16.57 | 0.0 | −2.9 | 2.9 | 0.988 | |
|  | Week 8 | 95 | 2 | 2.1 |  | 95 | 3 | 3.2 |  | 0.66 | 0.11 | 4.04 | −1.1 | −5.6 | 3.5 | 0.650 | |
| Total protein | Week 4 | 95 | 3 | 3.2 |  | 97 | 6 | 6.2 |  | 0.49 | 0.12 | 2.04 | −3.0 | −9.0 | 3.0 | 0.321 | |
|  | Week 8 | 95 | 4 | 4.2 |  | 95 | 12 | 12.6 |  | 0.30 | 0.09 | 0.98 | −8.4 | −16.3 | −0.5 | 0.037* | |
| Blood urea nitrogen | Week 4 | 95 | 8 | 8.4 |  | 97 | 4 | 4.1 |  | 2.14 | 0.62 | 7.35 | 4.3 | −2.6 | 11.1 | 0.219 | |
|  | Week 8 | 95 | 3 | 3.2 |  | 95 | 4 | 4.2 |  | 0.74 | 0.16 | 3.41 | −1.1 | −6.4 | 4.3 | 0.700 | |
| Creatinine | Week 4 | 95 | 6 | 6.3 |  | 97 | 4 | 4.1 |  | 1.57 | 0.43 | 5.74 | 2.2 | −4.1 | 8.5 | 0.494 | |
|  | Week 8 | 95 | 7 | 7.4 |  | 95 | 3 | 3.2 |  | 2.44 | 0.61 | 9.73 | 4.2 | −2.1 | 10.6 | 0.194 |  |
| Uric acid | Week 4 | 95 | 5 | 5.3 |  | 97 | 4 | 4.1 |  | 1.29 | 0.34 | 4.96 | 1.1 | −4.8 | 7.1 | 0.709 |  |
|  | Week 8 | 95 | 3 | 3.2 |  | 95 | 4 | 4.2 |  | 0.74 | 0.16 | 3.41 | −1.1 | −6.4 | 4.3 | 0.700 |  |
| Sodium | Week 4 | 95 | 1 | 1.1 |  | 97 | 4 | 4.1 |  | 0.25 | 0.03 | 2.25 | −3.1 | −7.6 | 1.4 | 0.182 |  |
|  | Week 8 | 95 | 0 | 0.0 |  | 95 | 1 | 1.1 |  | NA | NA | NA | −1.1 | −3.1 | 1.0 | 0.316 |  |

**Supplementary Table 8:** Participants showing out-of-range values after intervention (screening values normal) (continued)

| Item | Time point | *Euglena* group | | |  | Placebo group | | |  | Group difference | | | | | | |
| --- | --- | --- | --- | --- | --- | --- | --- | --- | --- | --- | --- | --- | --- | --- | --- | --- |
|  |  | *n* | Number of participants | Number of occurrences  (%) |  | *n* | Number of participants | Number of occurrences  (%) |  | ORs | 95% CIs− | 95% CIs+ | Δ (%) | 95% CIs− | 95% CIs+ | P value |
| Potassium | Week 4 | 95 | 3 | 3.2 |  | 97 | 7 | 7.2 |  | 0.42 | 0.11 | 1.67 | −4.1 | −10.3 | 2.2 | 0.206 |
|  | Week 8 | 95 | 2 | 2.1 |  | 95 | 4 | 4.2 |  | 0.49 | 0.09 | 2.74 | −2.1 | −7.1 | 2.9 | 0.407 |
| Chloride | Week 4 | 95 | 2 | 2.1 |  | 97 | 5 | 5.2 |  | 0.40 | 0.07 | 2.09 | −3.0 | −8.4 | 2.3 | 0.260 |
|  | Week 8 | 95 | 2 | 2.1 |  | 95 | 3 | 3.2 |  | 0.66 | 0.11 | 4.04 | −1.1 | −5.6 | 3.5 | 0.650 |
| Serum amylase | Week 4 | 95 | 0 | 0.0 |  | 97 | 8 | 8.2 |  | NA | NA | NA | −8.2 | −13.9 | −2.6 | 0.004** |
|  | Week 8 | 95 | 5 | 5.3 |  | 95 | 7 | 7.4 |  | 0.70 | 0.21 | 2.28 | −2.1 | −9.0 | 4.8 | 0.551 |
| Total cholesterol | Week 4 | 95 | 11 | 11.6 |  | 97 | 8 | 8.2 |  | 1.46 | 0.56 | 3.80 | 3.3 | −5.1 | 11.8 | 0.440 |
|  | Week 8 | 95 | 6 | 6.3 |  | 95 | 12 | 12.6 |  | 0.47 | 0.17 | 1.30 | −6.3 | −14.6 | 2.0 | 0.137 |
| HDL cholesterol | Week 4 | 95 | 5 | 5.3 |  | 97 | 3 | 3.1 |  | 1.74 | 0.40 | 7.50 | 2.2 | −3.5 | 7.8 | 0.452 |
|  | Week 8 | 95 | 7 | 7.4 |  | 95 | 2 | 2.1 |  | 3.70 | 0.75 | 18.29 | 5.3 | −0.8 | 11.3 | 0.088 |
| LDL cholesterol | Week 4 | 95 | 2 | 2.1 |  | 97 | 8 | 8.2 |  | 0.24 | 0.05 | 1.16 | −6.1 | −12.4 | 0.1 | 0.055 |
|  | Week 8 | 95 | 3 | 3.2 |  | 95 | 7 | 7.4 |  | 0.41 | 0.10 | 1.64 | −4.2 | −10.6 | 2.1 | 0.194 |
| Triglycerides | Week 4 | 95 | 9 | 9.5 |  | 97 | 7 | 7.2 |  | 1.35 | 0.48 | 3.77 | 2.3 | −5.6 | 10.1 | 0.572 |
|  | Week 8 | 95 | 5 | 5.3 |  | 95 | 7 | 7.4 |  | 0.70 | 0.21 | 2.28 | −2.1 | −9.0 | 4.8 | 0.551 |
| Glucose | Week 4 | 95 | 3 | 3.2 |  | 97 | 4 | 4.1 |  | 0.76 | 0.17 | 3.48 | −1.0 | −6.3 | 4.3 | 0.721 |
|  | Week 8 | 95 | 3 | 3.2 |  | 95 | 0 | 0.0 |  | NA | NA | NA | 3.2 | −0.4 | 6.7 | 0.081 |
| Hemoglobin A1c (NGSP) | Week 4 | 95 | 0 | 0.0 |  | 97 | 0 | 0.0 |  | NA | NA | NA | 0.0 | NA | NA | NA |
|  | Week 8 | 95 | 0 | 0.0 |  | 95 | 0 | 0.0 |  | NA | NA | NA | 0.0 | NA | NA | NA |

The number and proportion of applicable cases are reported, and between-group comparisons are presented as odds ratios (ORs) with 95% confidence intervals (CIs), along with differences in proportions (Δ) and their corresponding 95% CIs. Between-group comparisons were conducted using Pearson’s chi-square test.

Week 4, 4 weeks after intervention; Week 8, 8 weeks after intervention; NA, Not Available.

**P* < 0.05, ***P* < 0.01
